# Workplace-based learning in district health leadership and management strengthening: a framework synthesis

**DOI:** 10.1093/heapol/czae095

**Published:** 2024-10-09

**Authors:** Grace Kiarie, Lucy Gilson, Marsha Orgill

**Affiliations:** Division of Health Policy and Systems, School of Public Health, Faculty of Health Sciences, University of Cape Town, Falmouth Rd, Observatory, Cape Town 7925, South Africa; Division of Health Policy and Systems, School of Public Health, Faculty of Health Sciences, University of Cape Town, Falmouth Rd, Observatory, Cape Town 7925, South Africa; Department of Global Health and Development, London School of Hygiene and Tropical Medicine, Keppel Street, London WC1E 7HT, United Kingdom; The Children’s Institute, University of Cape Town, 46 Sawkins Road, Rondebosch, Cape Town 7700, South Africa

**Keywords:** Workplace-based learning, leadership, management, district health system, framework synthesis

## Abstract

Effective leadership and management has been identified as critical in enabling health systems to respond adequately to their population needs. The changing nature of low- and middle-income countries’ health systems, given resource scarcity, a high disease burden and other contextual challenges, has also led to learning—including workplace-based learning (WPBL)—being recognized as a key process supporting health system reform and transformation. This review used a framework synthesis approach in addressing the question: ‘What forms of WPBL, support leadership and management development; and how does such learning impact district health leadership and management strengthening?’. A search for English language empirical qualitative, mixed-methods and quantitative studies and grey literature published from January 1990 to May 2024 was conducted using four electronic databases (PubMed, EBSCOhost, Scopus and Web of Science). Twenty-five articles were included in the synthesis. The findings reveal that over the last decade, WPBL has received consideration as an approach for leadership and management development. While WPBL interventions differed in type and nature, as well as length of delivery, there was no conclusive evidence about which approach had a greater influence than others on strengthening district health leadership and management. However, the synthesis demonstrates the need for a focus on the sustainability and institutionalization of interventions, including the need to integrate WPBL interventions in health systems. To support sustainability and institutionalization, there should be flexibility in the design and delivery of such interventions and they are best supported through national or regional institutions.

Key messages

- Workplace-based learning (WPBL) has received growing consideration as an approach for leadership and management development in Africa.
- Context-specific learning, peer learning, the use of reflective practices and team-based learning were key forms of WPBL that contributed to district health leadership and management strengthening.
- For WPBL to impact district health leadership and management strengthening over the long term, sustaining and institutionalizing WPBL are important features to be considered.

## Introduction

Effective leadership and management has been identified as critical in enabling health systems to respond adequately to their population needs (Vriesendorp *et al*., 2010; Daire *et al*., 2014; Agyepong *et al*., 2018). For low- and middle-income countries (LMICs), leadership and governance challenges are particularly acute and are one of the stated reasons why LMICs did not achieve the Millennium Development Goals, alongside an under-skilled health workforce and the socio-economic determinants of health (Willis-Shattuck *et al*., 2008; Agyepong *et al*., 2018). Strengthening leadership and management has also been identified as important in supporting universal health coverage and improving health outcomes (Doherty *et al*., 2018; Martineau *et al*., 2018).

The COVID-19 pandemic, meanwhile, highlighted the need for more resilient LMIC health systems, demonstrating that learning is crucial for strengthening these systems and helping them to be better prepared to face future challenges (Sheikh *et al*., 2020; 2022). Considerable literature has been published on learning for health system reform and transformation in high-income countries (HICs), with workplace-based learning (WPBL) identified as vital to this process (Illeris, 2003; Manley *et al*., 2009; Manuti *et al*., 2015; Edmonstone, 2018). Some of the terms used for this type of learning include work-based learning, workplace learning and workplace-based learning (Illeris, 2003; Raelin, 2008; Doherty and Gilson, 2015). Consequently, WPBL can be a challenging concept to define and there is more than one definition (Matthews, 1999; Manuti *et al*., 2015). In this paper, we use the term workplace-based learning to mean learning that occurs in the workplace (Doherty and Gilson, 2015). The workplace can be a physical location but includes the shared values, ideas, actions and attitudes that define the working environment and network of relationships (Matthews, 1999). An employee could, therefore, work in another location, for example from home, but still consider themselves part of the workplace (Matthews, 1999). Learning that occurs in the workplace setting is referred to as onsite learning, while learning that occurs away from the workplace setting is referred to as offsite learning (Jacobs and Park, 2009). The learning that happens in the workplace can be informal, responding to critical changes or problems initiated within the workplace that require resolution, or formal, usually around more structured planned learning activities (Cunningham *et al*., 2004; Jacobs and Park, 2009; Doherty and Gilson, 2015; Manuti *et al*., 2015).

Conventional training programmes that focus on acquiring technical and operational skills through *ad hoc* and didactic educational methods in centralized locations away from the workplace have been shown to have limitations in strengthening health systems and improving health outcomes (Matovu *et al*., 2013; Edmonstone, 2018). While WPBL is not a magic bullet, it has the potential to contribute to changing health organizations by strengthening the capacity of health workers, improving productivity and users’ experiences and achieving continued transformation (Manley *et al*., 2009; Matovu *et al*., 2013). It also may offer potential or current leaders and managers, faced with an unrelenting pace of change, an opportunity to learn and reflect in the midst of work practice (Raelin, 2008; 2011).

In LMIC healthcare systems, leadership practice often adopts a top-down approach, driven by hierarchy, that has been shown to have limits in strengthening health systems and improving population health (Kwamie, 2015; Chunharas and Davies, 2016; Nzinga *et al*., 2018). National-level health leadership sets policies and the overall strategic direction for the health system and is influenced by the wider national and international environments (Gilson, 2012). The meso or organizational level comprises the local district, hospital and primary healthcare (PHC) managers and facility staff who convert national policies and any resources allocated to them into health services for the population (Daire *et al*., 2014; Bradley *et al*., 2015; Chunharas and Davies, 2016). The district health system (DHS) is often the key decentralized component and cornerstone of national health systems (Tarimo, 1991; Nzinga *et al*., 2021).

LMIC leadership and management development has to a large extent focused on course-based formal residential training programmes that build individual skills and capabilities for vertical disease programmes and/or lead to academic qualifications (Day, 2001; Daire *et al*., 2014; Edmonstone, 2018; Johnson *et al*., 2021). Furthermore, leadership and management development programmes have often taken trainees away from their workplaces for training, causing disruptions in health service delivery and involving only a few individuals from any facility or district (Matovu *et al*., 2013; Doherty and Gilson, 2015). A recent scoping review of leadership development programmes in sub-Saharan Africa showed that programmes were diverse in their design, learning content and teaching method, with no consistency in the conceptual approaches adopted or leadership frameworks used (Johnson *et al*., 2021). It was also observed that there was a lack of evidence about which format of leadership development programmes had more of an impact than others on health systems (Johnson *et al*., 2021).

No review has, to our knowledge, critically examined the available empirical work on WPBL and its contribution to strengthening leadership and management in LMICs at the district level. The aim of this study was to address this gap through a secondary analysis and synthesis of the literature about workplace-based approaches to leadership and management development and to identify whether they strengthened district health leadership and management. The review question was: ‘What forms of WPBL, support leadership and management development; and how does such learning impact district health leadership and management strengthening?’.

## Methods

### Synthesis method

The selection of the synthesis method was driven by the study’s purpose, which was both exploratory and explanatory. In other words, it sought to identify and explain experiences around the phenomenon of interest and generate new insights (Gilson, 2012). This phenomenon—WPBL strategies for leadership and management development—entails complex and multifaceted choices that require an understanding of context, policy processes, people and their relationships. Unlike the more structured review methods used to synthesize data concerning the effectiveness of interventions, framework synthesis offers the flexibility that is crucial for exploring such complex and context-dependent phenomena (Brunton *et al*., 2020). It addresses questions that go beyond ‘is it effective?’ and ‘what are people’s experiences?’ to include the issues of how something might work and under what circumstances (Langlois *et al*., 2018; Brunton *et al*., 2020). Framework synthesis was, therefore, particularly suited to our review question and our objective of exploring and analysing WPBL strategies.

### Review strategy

Following the usual practice in framework synthesis, the overall review question acted as a guide rather than the anchor of the study (Dixon-Woods, 2011; Langlois *et al*., 2018; Brunton *et al*., 2020). The synthesis was, therefore, undertaken in an iterative manner with each step building upon the previous one. Such synthesis begins with familiarization with the literature on the topic under study. This involved reviewing current theories and practices related to WPBL, as well as identifying relevant frameworks. Next, a suitable conceptual model is chosen to answer the review question. This model is then used as the basis for initial coding and theme generation. The framework acts as a scaffold against which findings from different studies can be brought together and organized (Oliver *et al*., 2008; Brunton *et al*., 2020). We found three frameworks on WPBL (Raelin, 1997; Matthews, 1999; Jacobs and Park, 2009), and after reviewing them, we selected the Matthews (1999) framework as it includes outputs and outcomes in which we were also interested. None of the articles included for synthesis applied the Matthews (1999) framework. Matthews (1999) notes that this framework seeks to describe a ‘holistic view of workplace learning’ and that while it may not answer questions on how to develop a WPBL environment, it does provide some useful factors to be considered in the delivery of WPBL. Here, we use this framework as a lens to understand how WPBL might lead to strengthening management and leadership in the DHS context. Key factors in the Matthews framework, as shown in Supplementary Appendix 4 in blue, include inputs (e.g. policies) interacting with individual and motivational issues, which lead to outputs (e.g. better competency) and outcomes (e.g. improved performance).

As the Matthews (1999) framework was developed for the private sector, additional concepts to reflect a public sector organizational context were added. These concepts are highlighted in red in Supplementary Appendix 4 and were drawn from the World Health Organization (WHO) framework for strengthening management at the district level; the Management Sciences for Health Leading, Managing, and Governing for Results Model; Jacobs and Park’s proposed conceptual framework of workplace learning; Manley *et al*.’s framework for work-based learning and WPBL tools and approaches from peer-reviewed literature and book chapters (Supplementary Appendix 4; Day, 2001; Cunningham *et al*., 2004; WHO, 2007; Raelin, 2008; Jacobs and Park, 2009; Manley *et al*., 2009; Vriesendorp *et al*., 2010; Doherty and Gilson, 2015). We also updated outputs and outcomes in the framework to reflect a public sector orientation on strengthened leadership and management (e.g. adding improved administrative efficiency of key resources, crucial management systems functioning and improved responsiveness to community needs; WHO, 2007).

We implemented several key steps to ensure the synthesis was conducted in a systematic way. The first step of the review process involved conducting a systematic search for research articles on WPBL and on district health leadership and management development programmes, using applicable keywords in the search strategy and choosing pertinent studies (Brunton *et al*., 2020). The keywords were derived from the review question and the conceptual framework, ensuring consistency throughout the review process. These included terms related to: (1) the type of learning such as ‘work-based learning’, ‘workplace learning’, ‘workplace based learning’, ‘action learning’, ‘mentoring’ and ‘coaching’; (2) the type of intervention such as ‘leadership’ and ‘management’ and (3) the sector in which the learning took place such as ‘health system’. Articles were carefully chosen using predetermined inclusion and exclusion criteria (Table 1). This ensured that only articles aligned with the review’s objective, question and theoretical underpinnings were included, which contributed to the overall rigour of the synthesis. EndNote reference manager was used to identify and remove duplicates, organize and store the references once the search was completed.

**Table 1.** Inclusion and exclusion criteria

Inclusion criteria	Exclusion criteria
Studies relevant to the research question	Studies not linked to WPBL or applicable to the research question
Empirical studies on WPBL linked to leadership and/or management development	Studies that are not based on empirical research
Studies that include qualitative, mixed-methods and quantitative methodology	Studies not published in English
Studies published in English	Studies not carried out in LMICs
Grey literature in English from credible sources like WHO or AHPSR	Studies published before the year 1990
Studies carried out in LMICs	
Studies carried out in PHC facilities and at the district level	
Original or review articles whose titles and abstracts include one or more of the key search terms	

The electronic bibliographic databases utilized for the search were Medline via PubMed, EBSCOhost [Academic Search Premier, Africa-Wide Information, Cumulative Index of Nursing and Allied Health Literature (CINAHL), Health Source: Nursing/Academic Edition and APA PsycInfo], Scopus and Web of Science. The database searches were limited to articles published after 1990, as it was in the 1990s that WPBL earned growing interest in HICs when Raelin’s critical framework was published (Raelin, 1997). Institutional databases like the WHO and the Alliance for Health Policy and Systems Research (AHPSR) were also included. Step two was to appraise the quality of the primary studies selected to determine which ones will be used in the synthesis (Brunton *et al*., 2020). Thirdly, we conducted data extraction, which was guided by the conceptual framework. Using a conceptual framework provided a systematic approach to data extraction, ensuring that data were consistently cross-checked against the framework for relevance (Barnett-Page and Thomas, 2009; Dixon-Woods, 2011; Brunton *et al*., 2020). Qualitative data were extracted into a data extraction table (Supplementary Appendix 3). Finally, interpretation and synthesis of the extracted data were undertaken. The framework enabled a more transparent and rigorous synthesis by guiding how the data were categorized and analysed based on key concepts (Barnett-Page and Thomas, 2009; Dixon-Woods, 2011; Brunton *et al*., 2020). To ensure transparency in presenting the evidence, we adhered to the Preferred Reporting Items for Systematic Reviews and Meta-Analyses statement (Page *et al*., 2021).

In this review, the primary author screened the abstracts and full articles. The screening decision was presented to the second and third authors for review, and consensus was reached. The primary author extracted data from the eligible articles, which was then reviewed and commented on by the second and third authors in regular team meetings and draft working documents. The primary author wrote four draft syntheses, which the second and third authors conducted ongoing reviews on and provided critical comments, further interpreting the results.

### Quality appraisal

We adopted an approach of assessing the quality of articles while in the process of building the synthesis, rather than excluding articles before the synthesis process, to avoid excluding any ‘nuggets of wisdom’ in seemingly methodologically weak studies (Dixon-Woods *et al*., 2004; 2006; 2007; Pawson, 2006). In this review, the primary author made quality judgements and the other authors verified key appraisal points.

### Data synthesis

Following the framework synthesis approach, the conceptual framework provided a structured way to code, organize and analyse extracted data (Matthews, 1999; Barnett-Page and Thomas, 2009). If relevant data from the included studies did not fit the pre-existing categories in the first round of coding, secondary thematic coding allowed these data to be considered (Dixon-Woods, 2011). We examined all codes to identify associations or patterns across papers. We then identified notable themes within categories, such as key similarities or differences in motivation among the papers (Pope *et al*., 2000; Brunton *et al*., 2020). An interpretation of the relationships between themes is offered to explain the main findings (Pope *et al*., 2000; Brunton *et al*., 2020). This process therefore moved analysis to synthesis—that is, from merely merging and amalgamating data to a greater level of abstraction, generating new ideas and insights (Pope *et al*., 2000; Dixon-Woods *et al*., 2005; 2006; Gilson, 2014).

We use the framework by Matthews (1999), including our public sector additions, to understand how inputs for WPBL interact with individual and motivational issues to achieve proximal outputs. Finally, we consider whether WPBL leads to outcomes—while also acknowledging the effect of environmental influences and organizational characteristics on the process.

## Results

**Figure 1.**
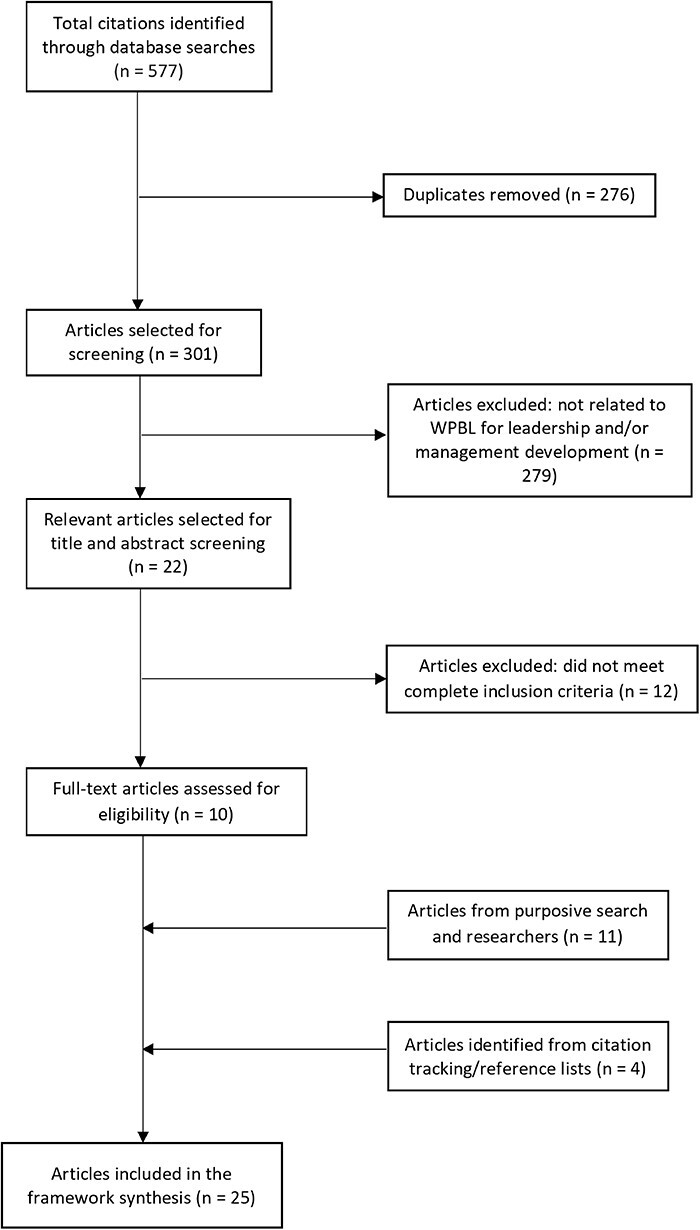
Flow diagram of articles included in the search process

The literature search found 577 articles. After the removal of duplicates, 301 articles remained, of which 279 were excluded as not related to WPBL for leadership and/or management development. Twenty-two articles were found to be potentially relevant. The titles and abstracts of these articles were screened against the predetermined inclusion and exclusion criteria. After full-text reading, only 10 articles met the full inclusion criteria. Due to the limited evidence on WPBL for district health leadership and management development, a purposive search for articles on leadership and management development programmes in district health systems was conducted. This search identified 10 relevant articles. Citation tracking using the reference lists of included articles identified other pertinent studies. Additional insights on empirical studies to include in the review were obtained from experienced health policy and systems researchers. In total, 25 articles were included in the framework synthesis. Figure 1 outlines the number of articles included at each stage of the search process.

### Characteristics of the included studies

Of the 25 included articles, 22 were published between 2011 and 2022, with none published before 2002, although the literature search strategy included articles published from the year 1990. The included articles covered research studies that had a WPBL focus, reporting on WPBL interventions in 11 countries (Table 2). The majority of the included studies were conducted in Africa. Four articles from Kenya covered the same WPBL intervention as did two articles from India, and all these articles had the same first author across Kenya and India. One article covered a multi-country study in Ghana, Tanzania and Uganda (Martineau *et al*., 2018). It was notable that we did not find any papers from the regions of Eastern Europe, South America, Central Africa and South East Asia, although this could have been an oversight of our search strategies.

**Table 2.** Number of articles from each country

Country	Number of articles
Kenya	6
South Africa	4
Uganda	4
Mozambique	2
Zambia	2
India	2
Ghana	2
Liberia	1
Tanzania	2
Egypt	1
Ethiopia	1

The papers utilizing only qualitative methods (*n* = 13) generally provided thick descriptions of the intervention and context, and we were able to draw more data from the experiences being described (Table 3; Tolley *et al*., 2004). One of the qualitative studies was not based on an explicit WPBL intervention; however, WPBL was observed while conducting research on district financial management and was documented by the author (Choonara *et al*., 2017). A table containing a summary of the included articles is provided in Supplementary Appendix 2. This table describes the type and nature of the WPBL intervention in each paper, including the length of delivery of each intervention, and whether an official qualification was conferred upon successful completion. In addition, the table in Supplementary Appendix 2 provides information on the aim of each paper, the stakeholders driving the interventions, the methodology used, the WPBL participants and the intervention setting and summarizes the findings from each paper.

**Table 3.** Types of methodologies utilized in the included studies

Study methodology	Number of studies
Qualitative study^a^	13
Mixed-method study	4
Quasi-experimental study	4
Quantitative study	4

a In this review, a qualitative study was a study that only employed qualitative methods and data were collected through interviews, participant and non-participant observations, focus groups and document reviews (Tolley *et al*., 2004).

### Characteristics of the WPBL interventions

All included articles offer insights on workplace-based leadership and management development programmes being carried out at the district level in LMICs. All the papers, except Choonara *et al*.’s (2017) study, reported a structured systematic approach to the design and delivery of the WPBL intervention, though with considerable diversity in the approaches utilized. Most of the WPBL interventions were practice-based (*n* = 15), while the rest were rooted in a hybrid model (*n* = 10) that included a mixture of both course- and practice-based approaches. The course-based approach is characterized by leadership and management being taught through formal educational training modules, delivered by management consultants or academicians and often resulting in an academic qualification like a diploma or master’s degree (Daire *et al*., 2014; Doherty and Gilson, 2015; Edmonstone, 2015; 2018). The practice-based approach is characterized by leadership and management development being facilitated through problem-solving and reflection in the workplace. Academic qualifications are not the key goal of practice-based learning, but instead the focus is on participatory capacity building and collaborative team learning (Daire *et al*., 2014; Edmonstone, 2015; Doherty *et al*., 2018).

The majority of the interventions (*n* = 17) were driven by country-level stakeholders^1^ (Table 4), but three were led by external actors^2^ (Sherr *et al*., 2013; Edwards *et al*., 2015; Desta *et al*., 2020). Where there were collaborations among stakeholders, especially between country and external actors (*n* = 7), it was difficult to determine which stakeholders were driving the WBPL interventions, as this information was not always explicitly stated in each article.

**Table 4.** Stakeholders driving WPBL interventions

Stakeholders driving WPBL	Number of interventions
Country-level higher education institutions	10
Country-level research institutions	2
Other country-level actors	5
External actors and/donors	3
Collaborations between country-level actors and external actors/donors	7

Twelve of the WPBL interventions were between 6 months and 2 years in length. Longer interventions were embedded in research projects or other types of capacity development programmes. For example, Sherr *et al*. (2013) describe a WPBL intervention embedded in the 7-year Mozambique Population Health Implementation and Training partnership project. The interventions described by Cleary *et al*. (2018a) in South Africa, Martineau *et al*. (2018) in Ghana, Tanzania and Uganda and Tetui *et al*. (2017) in Uganda were between 2 and 5 years in length and embedded in broader action research projects. In three other interventions, WBPL formed part of formal educational programmes accredited by higher education institutions. These programmes ranged from 1 to 2 years in length, and an official qualification was conferred upon successful completion (Dovey, 2002; Doherty *et al*., 2018; Foster *et al*., 2018). As mentioned previously, one paper was not based on an explicit intervention, but WPBL was observed while research on district financial management was taking place (Choonara *et al*., 2017). The majority of the interventions (*n* = 14) included district health managers or members of District Health Management Teams (DHMTs; Table 5). Some interventions included facility managers (*n* = 13); members of Sub-District Management Teams (SDMTs; *n* = 4); and health workers (*n* = 5). The intervention in India also included politicians (Prashanth *et al*., 2014a).

**Table 5.** Participants involved in WPBL interventions

WPBL participants	Number of interventions
District managers or DHMT(s)	14
Facility managers	13
SDMT(s)	4
Health workers	5
Elected members of local government	1
Interdisciplinary participants involved in district financial management	1
District Human Resources for Health teams	1
District Hospital Management Team	1
Other staff in PHC facilities	1
Provincial (governorate/regional) managers	2

### WPBL supporting leadership and management development

The following section summarizes and analyses the evidence to provide an understanding of WPBL leadership and management development programmes in the DHS and its outcomes of leadership and management strengthening. The findings are presented below according to the key topic headings in the conceptual framework. As none of the included studies applied the Matthews (1999) framework, data did not fit neatly into its categories and so an interpretive approach was used when extracting data. Each category will be considered individually and a summary of the pertinent findings will be provided. Relationships between themes will be offered to explain the key ideas emerging from the evidence.

### Inputs

Inputs are the variety of mechanisms that contribute to the design and delivery of WPBL interventions (Matthews, 1999). Matthews (1999) identified the need for strategies and activities as important inputs for WPBL—and those identified in this research, as shown in Table 6, are supported by the broader WPBL literature (Day, 2001; Cunningham *et al*., 2004; Raelin, 2008; Doherty and Gilson, 2015).

**Table 6.** Inputs of WPBL interventions utilized for leadership and management development

WPBL inputs	Number of interventions
Facilitators	19
Action projects	10
Mentoring	10
Coaching	9
Peer learning	7
Action learning	7
Reflection	6
Case studies	3

#### The use of facilitators

In principle, facilitators can assist participants in the process of transforming themselves, their teams, their workplaces and their communities (Manley *et al*., 2009). All the interventions, except two (Edwards *et al*., 2015; Choonara *et al*., 2017), reported using facilitators. Facilitators were actively involved during workshops, classroom modules, meetings, coaching sessions, reflection sessions and the coordination and implementation of action projects. Several studies encouraged former participants to take on these roles for future iterations of the WPBL intervention (Mansour *et al*., 2010; Rowe *et al*., 2010; Matovu *et al*., 2013; Chelagat *et al*., 2019).

#### Action projects

Action projects are specific work assignments, created and implemented by participants of training programmes, that have strategic value to an organization. The assignments often involve improving services at the workplace over a period of time (Cunningham *et al*., 2004; Raelin, 2008; Doherty and Gilson, 2015). Key to the success of WPBL interventions was matching participants to action projects that were equally valuable to both the participants and the health institutions (Nakanjako *et al*., 2015). Four studies explored action projects linked to maternal and neonatal or child health issues (Mansour *et al*., 2010; Kwamie *et al*., 2014; Tetui *et al*., 2017; Desta *et al*., 2020). While the action projects in two of the studies looked at issues around human immunodeficiency virus/acquired immunodeficiency syndrome (HIV/AIDS; Nakanjako *et al*., 2015; Foster *et al*., 2018). The rest of the action projects in the included studies were varied in the issues they tackled.

#### Action learning

Closely linked to action projects was the use of action learning to facilitate WPBL. Action learning is a series of activities where people who work together generate learning through engagement in solving a problem at work. Learning occurs as they address these problems, take actions to resolve them and put the solutions into practice (Day, 2001; Cunningham *et al*., 2004; Raelin, 2008; Doherty and Gilson, 2015). Unlike action projects, action learning does not require the creation of new projects to solve workplace challenges (Doherty and Gilson, 2015). The District Innovation and Action Learning for Health Systems Development (DIALHS) project found that its action learning strategy facilitated a strengthening of distributed and relational leadership, by adopting a team approach to learning across two different layers of the health system, within a comparatively resource-poor sub-district in South Africa (Cleary *et al*., 2018a). The idea of distributed and relational leadership refers to leadership that is non-hierarchal and collaborative, engages people and is associated with building trust and empowerment (Cleary *et al*., 2018a). Though time-intensive, the strength of action learning as a WPBL approach is that, like action projects, it is action oriented and tied to organizational priorities (Day, 2001).

#### Mentoring

Mentoring is a strategic relational approach within an organization that over a period of time supports people in their career development as they interact with a more experienced person (Day, 2001; Cunningham *et al*., 2004; Doherty and Gilson, 2015). Mentorship played a crucial part in the WPBL interventions in India and Zambia, with participants being assigned mentors to follow them throughout the duration of the intervention, helping them to problem solve and apply lessons learnt to their workplaces (Prashanth *et al*., 2014b; Mutale *et al*., 2017). In Mozambique, a health management mentoring initiative supported mentors to spend time with managers in their workplaces, and to support them with their day-to-day challenges around supply chain management, accounting, Human Resources for Health and transportation (Edwards *et al*., 2015).

#### Coaching

Coaching is a peer relationship that consists of one-on-one learning between a coach and a client, around practical goal-focused areas of personal development and behaviour modification that improve the client’s performance (Day, 2001; Cunningham *et al*., 2004; Doherty and Gilson, 2015). While a mentor is a more experienced person who provides technical advice to a mentee, a coach may not have the technical skill, but rather someone who is skilled at listening to a client and helping them to explore the issues they face in greater depth (Doherty and Gilson, 2015). Through a series of engagements over a period of time between a research team (the coaches) and PHC facility managers together with an SDMT (the clients), coaching emerged as an approach for a WPBL intervention in South Africa (Cleary *et al*., 2018a). Coaching was focused on ‘creating a community of practice’ that incorporated developing ‘relational leadership skills’ (Cleary *et al*., 2018a). District and facility managers reported a greater appreciation for relational leadership, which led to better cohesion among workplace teams (Cleary *et al*., 2018a).

#### Peer learning

Studies in South Africa, Zambia, Ghana, Tanzania and Uganda reported that peer learning provided support, allowed the sharing of ideas and best practices, enabled skills transfer and ensured relevance to the solutions selected for health system challenges (Doherty *et al*., 2018; Foster *et al*., 2018; Martineau *et al*., 2018; Cleary *et al*., 2018a). Peer learning strengthened a community of practice among facility managers and enabled them to gain confidence in their own ability as leaders through the support they received from one another (Foster *et al*., 2018).

#### Reflection

Reflection is the practice of stepping back from one’s experience to ponder and think through what is happening to oneself and/or to others. Creating new meaning and expressing this new understanding is the product of reflective learning practices and often provides the foundation for future action (Cunningham *et al*., 2004; Raelin, 2008; Doherty and Gilson, 2015). Collaborative reflective practices between WPBL implementing teams and participants in Kenya and South Africa focused on personal values and relationships in the workplace (Cleary *et al*., 2018a; Nzinga *et al*., 2021). Reflection was vital for learning from experience and refining knowledge learnt prior to further action (Dovey, 2002; Martineau *et al*., 2018; Cleary *et al*., 2018a).

#### Case studies

During classroom modules, real-world case studies about understanding and navigating complex health systems were sometimes used as a teaching aid to connect theory to practice and to facilitate the development of leadership skills needed to address health system challenges (McLean, 2016; Doherty *et al*., 2018; Nzinga *et al*., 2021). In Kenya and South Africa, in-depth discussions of cases among health managers in the classroom facilitated learning about the ‘how to’ of management and leadership through examples (Doherty *et al*., 2018; Chelagat *et al*., 2019; Nzinga *et al*., 2021).

### Organizational characteristics

In this review, organizational characteristics refer to the culture, learning environment and supportive infrastructure within the organization that would support the WPBL (Matthews, 1999; Manley *et al*., 2009).

#### Opportunities in the organization that support WPBL

Health managers in Ghana, Tanzania and Uganda appreciated the organizations’ commitment to support the managers’ WPBL by providing the managers time for the learning process. Workshops, inter-district meetings and protected time gave managers the opportunities to work through learning activities and complete action projects (Nakanjako *et al*., 2015; Martineau *et al*., 2018). Across several studies in South Africa, Uganda, Zambia and Mozambique, organizational engagement with key health system stakeholders during the WPBL intervention, for example engagement with health worker unions, was an additional health system enabler that supported WPBL (Dovey, 2002; Gormley and McCaffery, 2013; Sherr *et al*., 2013; Edwards *et al*., 2015; Nakanjako *et al*., 2015; Foster *et al*., 2018). This facilitated trust among multiple stakeholders and the acceptance of the intervention by health system actors (Cleary *et al*., 2018b). Similarly, meaningful partnerships built over time, for example between universities and the health system, were fruitful in enabling the successful implementation of WPBL (Lehmann and Gilson, 2015; Cleary *et al*., 2018a).

#### Challenges in the organizational environment for WPBL

Several of the studies in Zambia, Ghana, Uganda and Liberia reported that district-level health system managers were often professional healthcare workers, such as clinicians and nurses, who were promoted to management positions due to clinical experience and not necessarily leadership or managerial training or experience. This meant they were also still drawn on for clinical work while also trying to attend leadership training and development (Rowe *et al*., 2010; Kwamie, 2015; Nakanjako *et al*., 2015; Mutale *et al*., 2017; Foster *et al*., 2018). As a result, many of the managers had heavy workloads, performing clinical duties and administrative tasks, which meant participation was affected (Sherr *et al*., 2013; Tetui *et al*., 2017; Doherty *et al*., 2018; Foster *et al*., 2018). In South Africa, managers’ workloads proved to be a constraint for some in completing the leadership training programme. Furthermore, heavy workloads made networking and mentorship difficult to achieve (Doherty *et al*., 2018).

Structural constraints, such as centralization of bureaucratic processes and lengthy bureaucratic mechanisms to ensure accountability, led to difficulty in accessing financial resources and delayed procurement of needed resources such as IT equipment and internet connectivity, which impeded the implementation of WPBL interventions, despite health system managers’ intentions to actively participate in the learning (Choonara *et al*., 2017; Cleary *et al*., 2018a). Similarly, across the WPBL interventions in Zambia, India, Uganda, Kenya, Tanzania, Ghana and Mozambique, budget constraints, under-staffed facilities, limited access to technology, high turnover of health managers and healthcare workers, recurrent stock out of medicines and supplies and poor infrastructure, were factors that negatively influenced the workplace learning process (Gormley and McCaffery, 2013; Sherr *et al*., 2013; Kwamie *et al*., 2014; Prashanth *et al*., 2014b; Edwards *et al*., 2015; Tetui *et al*., 2017; Foster *et al*., 2018; Chelagat *et al*., 2020; 2021b; Tomblin Murphy *et al*., 2022).

Several studies found that organizational culture limited the gains that could be realized by WPBL interventions. In South Africa, resistance to change from managers and staff who had not undergone the leadership training programme were constraints on WPBL (Doherty *et al*., 2018). Difficult workplace dynamics due to power imbalances and a silo mentality among staff in different departments, meanwhile, limited action learning processes. In addition, workplace line managers were not always supportive of participants’ needs to practice new skills acquired or allowed flexibility for participants to make mistakes (Doherty *et al*., 2018). In Kenya, poor management support, as well as a deeply rooted culture of not questioning authority on what needs to be given precedence in the health system, was a hindrance to the transfer of learned leadership knowledge to the workplace (Chelagat *et al*., 2019; Nzinga *et al*., 2021).

### Individual and motivational issues

#### General motivation in the workplace was strongly linked to organizational culture

Across the included studies, low health worker motivation was a common theme that negatively impacted the implementation of WPBL interventions. Studies in Uganda, Ghana, Egypt and Mozambique reported poor staff attitudes and low morale among health workers and managers (Mansour *et al*., 2010; Kwamie *et al*., 2014; Edwards *et al*., 2015; Tetui *et al*., 2017). Frontline health workers and district health managers had become increasingly apathetic and lacked the desire to change things (Prashanth *et al*., 2014b; Nzinga *et al*., 2021). Health workers were rarely at their workstations and had a low commitment to health service quality (Tetui *et al*., 2017). Additionally, facility managers were not always trusted by senior-level management to make decisions based on their local context and felt powerless to impact their environment (Dovey, 2002; Cleary *et al*., 2018a).

#### Motivation directly linked to WPBL activities

However, the structure, delivery and perceived benefit of WPBL was a key motivator for health managers. Participants appreciated leadership development programmes that were short, focusing on a few vital skills with practical tools; fitted in with work schedules; involved different health system stakeholders and allowed for peer learning (Rowe *et al*., 2010; Tetui *et al*., 2017; Martineau *et al*., 2018). In South Africa, participants in the broader DIALHS collaboration appreciated the balance of both theoretical and practical activities geared towards solving workplace challenges (Doherty *et al*., 2018). In Egypt and Uganda, the WPBL hands-on approach promoted participants’ ownership of local health systems and provided practical tools to address everyday health system challenges (Mansour *et al*., 2010; Tetui *et al*., 2017). In Zambia, facility nurse managers were motivated to pursue WPBL to earn Continuous Professional Development points needed to fulfil relicensure requirements and earn the title of ‘head nurse in charge’ on completion of training (Foster *et al*., 2018).

### Environmental influences

The wider political, societal and economic environment influenced organizational context and consequently WPBL.

#### Opportunities

Government policies and strategic initiatives were factors that enabled the adoption of WPBL. In Zambia, the Ministry of Health (MOH) developed a Governance and Management Capacity Building Strategic Plan whose primary goal was to improve health system governance (Mutale *et al*., 2017). This led to the implementation of a WPBL intervention known as the Zambia Management and Leadership Academy (ZMLA) (Mutale *et al*., 2017). Additional studies in Uganda, Zambia, Liberia and Ethiopia reported that a key driver for the implementation of leadership and management development programmes was whether they were introduced, led or endorsed by the MOH, or complimented MOH guidelines and existing trainings (Rowe *et al*., 2010; Gormley and McCaffery, 2013; Foster *et al*., 2018; Desta *et al*., 2020).

#### Challenges

Several studies reported that the broader hierarchical governance structure restricted the impact of WPBL interventions. In India, health managers expressed frustration at the ‘lack of power to make changes at the taluka and district level’, despite the decentralized planning brought about by the National Rural Health Mission policy initiative (Prashanth *et al*., 2014b). In Mozambique, decentralization had been slow and uneven, and it was unclear which health system activities were considered under the scope of the district, province or national level. As such, it was difficult to define the role and thus strengthen the capacity of district managers (Sherr *et al*., 2013; Edwards *et al*., 2015). Furthermore, political interference negatively influenced WPBL interventions. In Kenya and South Africa, managers reported that constant political interference undermined health system innovation and threatened managers’ job security, such that decentralization had minimal impact on increasing local authority, planning and decision-making (Doherty *et al*., 2018; Nzinga *et al*., 2021). Finally, it is important to take into account the impact of wider economic conditions and technological development on the delivery of WPBL in LMICs. Studies in India, Egypt, Mozambique and Uganda reported that in poor rural areas, resource constraints made it difficult to implement WPBL interventions (Mansour *et al*., 2010; Sherr *et al*., 2013; Prashanth *et al*., 2014b; Edwards *et al*., 2015; Tetui *et al*., 2017).

### Outputs

Outputs are the immediate tangible results that are observed in the workplace after WPBL (Matthews, 1999).

#### Positive experiences

Several studies reported that as a result of WPBL, there was improvement in team dynamics resulting in mutual trust and respect among team members. Team dynamics was an output identified as part of our adaptation to the original Matthews (1999) framework as shown in Supplementary Appendix 4. The four studies in South Africa reported that after the WPBL interventions, there was an improvement in teamwork that led to the formation of cohesive teams (Dovey, 2002; Choonara *et al*., 2017; Doherty *et al*., 2018; Cleary *et al*., 2018a). Better trust and harmony among the SDMTs as well as within relationships between facility managers and staff were observed in the Mitchells Plain sub-district. This trust led to facility managers being given more discretionary decision space (Cleary *et al*., 2018a). Additionally, managers understood their roles and personal strengths and weaknesses and were, thus, able to transform interpersonal relationships with staff, supervisors and stakeholders (Cleary *et al*., 2018a). In Zambia and Tanzania, the joint participation of facility heads and district managers improved their relationships and the oversight and accountability for community health (Foster *et al*., 2018; Tomblin Murphy *et al*., 2022). With a shared vision, teamwork and coordination of work activities improved (Mutale *et al*., 2017; Tomblin Murphy *et al*., 2022).

The multi-country study in Ghana, Uganda and Tanzania and a study in Mozambique reported an improvement in work climate with a transformation in the way DHMTs functioned, as they began taking ownership of health system challenges and taking the initiative to solve these problems (Sherr *et al*., 2013; Martineau *et al*., 2018). Through applying leadership and management practices, DHMTs in Egypt, Uganda, Mozambique and Zambia learnt to work together and were empowered to actively engage and mobilize community stakeholders to address public health problems (Mansour *et al*., 2010; Sherr *et al*., 2013; Tetui *et al*., 2017; Foster *et al*., 2018). Stakeholder collaborations increased managers’ awareness of and adaptability to community needs. With improved trust among stakeholders, partnership and commitment towards new ideas of leadership and management practices were initiated and sustained (Tetui *et al*., 2017).

#### Negative experiences

In India, poor teamwork persisted among interdisciplinary district team members because of prevailing socio-cultural values where doctors were automatically viewed as team leaders (Prashanth *et al*., 2014b). Team power dynamics determined whether non-medical members’ contributions to improving organizational performance were considered (Prashanth *et al*., 2014b). In Ghana, meanwhile, initial appreciation of the WPBL intervention due to its novelty wore off over time, as it was resource intensive and exacerbated managers’ time and resource constraints (Kwamie *et al*., 2014). For instance, trying to involve additional staff to help in WPBL intervention activities, in particularly understaffed districts, without disrupting service delivery proved difficult (Kwamie *et al*., 2014).

### Outcomes

In principle, outcomes refer to the end results or consequences of WPBL and, ideally, should nurture the continued growth and development of an employee and the organization (Matthews, 1999; Manley *et al*., 2009). In this section, we show the outcomes as reflected in the different studies. Not all papers refer to these as outcomes, but we have imposed the adapted framework on the data.

#### Positive

A stated outcome for many of the WPBL interventions was improved health service delivery or health system performance. In Kenya, health managers made positive impacts on health system performance and efficiency indicators, including increase in skilled birth attendance, full child immunizations, utilization of in- and out-patient services and reduced out-patient turnaround times, through the action projects they engaged in (Chelagat *et al*., 2020; 2021b). In Egypt, there was an increase in prenatal care utilization, number of new family planning visits and use of contraceptives, which reduced fertility rates (Mansour *et al*., 2010). A reduction in the maternal mortality rate was also reported, 2 years after all the PHC facilities had gone through the leadership development programme for health workers (Mansour *et al*., 2010). While in Uganda, there was an improvement in HIV/AIDS health service delivery, construction of needed health facility infrastructure and ambulances for health facilities were acquired (Nakanjako *et al*., 2015; Tetui *et al*., 2017).

Two studies in South Africa reported that wider organizational learning was realized because of workplace leadership development interventions, which we also understood to reflect strengthened leadership and management (Dovey, 2002; Choonara *et al*., 2017). As a result of a district financial management team developing agency to address DHS constraints, the broader district staff were motivated to become solution driven (Choonara *et al*., 2017). Additionally, team members were able to generate and share new knowledge to enhance the collective knowledge within the district (Choonara *et al*., 2017). In Mozambique, meanwhile, there was an improvement in forecasting for health system needs, with overall progress in coordination and planning within the province, and greater transparency of accounting practices (Sherr *et al*., 2013; Edwards *et al*., 2015). This was greatly influenced by the historical partnerships among stakeholders, which had established trust and taking ownership of health system challenges (Sherr *et al*., 2013).

In South Africa and Zambia, managers remained committed to their roles in the public health system and within the same provinces after the health leadership training (Mutale *et al*., 2017; Doherty *et al*., 2018). This continuity in their positions had the capability to positively impact the health system by using their new leadership skills towards solving health system problems (Mutale *et al*., 2017; Doherty *et al*., 2018). Similarly in Uganda, all programme graduates stayed in health leadership positions within the country (Nakanjako *et al*., 2015). As a result of WPBL interventions, fellows were able to make recommendations to inform HIV policy formation and review procedures and health worker training for the national HIV/AIDS programme (Nakanjako *et al*., 2015).

#### Negative

Three studies reported that some participants did not complete the workplace-based leadership and management development programmes. In Kenya, only 8 out of the 30 participants went through the full training (Nzinga *et al*., 2021). In Uganda, one fellow did not complete the Afya Bora fellowship programme due to personal reasons (Nakanjako *et al*., 2015). While in Zambia, two facility managers did not complete the leadership and management programme (Foster *et al*., 2018). Another study in Zambia reported that many trainees did not implement the solutions they generated to solve workplace problems, as graduation from ZMLA did not depend on this (Mutale *et al*., 2017).

Several studies reported that the hierarchical governance structure found in the health system remained unchanged. In South Africa, the broader hierarchical governance context put DHMTs’ capacity gains at risk, as senior/higher-level managers had not undergone the workplace-based leadership and management development programme themselves (Dovey, 2002). In Ghana, the top-down manner in which the leadership development programme was introduced reinforced hierarchical and highly centralized decision-making processes. There was no change in relationships between the district and regional levels (Kwamie *et al*., 2014). In Kenya, providing feedback to superiors continued to be a problem as it was not appreciated (Nzinga *et al*., 2021).

### Sustainability and institutionalization

Inductively, we also identified two additional topics we feel could be included in the adapted framework for WPBL. The importance of the sustainability and institutionalization of WPBL interventions were the two new and recurring themes that emerged during data extraction.

#### Sustainability

Sustainability refers to the continued use of interventions after they have been adopted and implemented within an organization (Novotná *et al*., 2012). Long-term partnerships between health system managers and key stakeholders in Mozambique, Kenya and South Africa were reported to be beneficial in sustaining WPBL (Sherr *et al*., 2013; Cleary *et al*., 2018a; Nzinga *et al*., 2021). These partnerships improved trust, and commitment towards new ideas was initiated and sustained. Another study in South Africa reported that the sustainability of workplace projects was maintained, and these projects continued in successive cycles of strategic action, as a new member of the DHMT enrolled in the district health leadership programme in each successive cycle (Dovey, 2002). Teams completing projects demonstrated a sustained culture of collaborative action, with improvement in strategic thinking and how to manage power dynamics in the workplace (Dovey, 2002).

However, questions about the sustainability of WPBL interventions arose as a number of the reported interventions were donor funded. Donor funding tends to be an unpredictable source of long-term funding (Johnson *et al*., 2021). In several cases, interventions had to make adjustments to the design and delivery of WPBL due to changes in donor funds and priorities, while in some countries there were no additional iterations of the intervention once donor funding ended (Sherr *et al*., 2013; Edwards *et al*., 2015; Kwamie, 2015). In contrast, the multi-country study (Ghana, Uganda and Tanzania) reported that external funding for the implementation of WPBL interventions was deliberately not provided to instil an entrepreneurial approach to resource mobilization and not jeopardize sustainability, commonly seen once externally funded projects end (Martineau *et al*., 2018). This was seen as a risk. However, DHMTs were informed of the lack of funding prior to agreeing to be involved in the WPBL interventions. Some DHMTs saw this as part of the learning process on how to better utilize available resources (Martineau *et al*., 2018).

To ensure sustainability, several programmes remained flexible during the design and delivery of WPBL. Shifts in national programmes and available funding in Mozambique required adaptations (flexibility) to the management training in Sofala Province. This led to more of a focus on mentoring managers (on formulating data-driven decisions), over managers attending in-service training courses (Sherr *et al*., 2013). Furthermore, to build sustainability into WPBL, it was important to ensure the learning content was specific to the local contexts to foster creativity and innovative solutions for specific problems in a particular setting (Matovu *et al*., 2013; Doherty and Gilson, 2015; Edmonstone, 2015; Cleary *et al*., 2018a; Johnson *et al*., 2021). In Egypt, the WPBL intervention was taught in Arabic and the MOH facilitators reviewed the intervention exercises to ensure they were context-specific (Mansour *et al*., 2010).

#### Institutionalization

Institutionalization can be defined as ‘staying power’ or endurance of change that becomes integrated in the regular routines and daily practices within an organization (Novotná *et al*., 2012). As such institutionalization is seen as a social phenomenon that is cultivated through shared experiences (Koon *et al*., 2020). When institutionalized, organizational practices become deeply engrained within their environments to bring about significant change in the approach organizations use to deliver services (Novotná *et al*., 2012; Johnson *et al*., 2021). Embedding interventions within the health system ensured institutionalization of WPBL (Cleary *et al*., 2018b; Johnson *et al*., 2021). In Mozambique, this led to the WPBL intervention being deeply integrated within the Sofala provincial health department and its 11 district health systems. Focus on the entire province ensured longevity of district capacity gains as well as what would be suitable for national scale-up (Sherr *et al*., 2013). Closely linked to embeddedness in the health system was the idea of country ownership. As WPBL met country needs, it was embraced by relevant health system actors in Zambia (Foster *et al*., 2018). With this in mind, alignment with national and sub-national goals or wider health system administrative and governance processes was important to institutionalizing WPBL interventions (Dovey, 2002; Prashanth *et al*., 2014b; Nakanjako *et al*., 2015; Mutale *et al*., 2017; Foster *et al*., 2018; Desta *et al*., 2020; Johnson *et al*., 2021; Chelagat *et al*., 2021a).

Offering workplace learning through national or regional institutions ensures that WPBL is institutionalized within the health system (Johnson *et al*., 2021). Engagement with the University of Zambia (UNZA) and the Zambian Union of Nurses Organization in a WPBL intervention for nurses leading PHC teams led to UNZA’s School of Nursing beginning to include the intervention’s leadership and management elements in the nursing pre-service training curriculum (Foster *et al*., 2018). Accreditation of WPBL provided an incentive for health system actors to participate in it, further encouraging its integration into health system practices (Mutale *et al*., 2017; Johnson *et al*., 2021). After ZMLA received the National Institute for Public Administration certification, training became popular among busy health workers in Zambia. Managers could not only use the skills gained towards solving current health system challenges but also use their diplomas to apply for promotions or other employment within the health system (Mutale *et al*., 2017).

## Discussion

This review used the framework approach to synthesize evidence on WPBL, specifically to identify different forms of WPBL, derive lessons from these experiences for leadership and management development in the context of a DHS and understand outputs achieved as well as outcomes on leadership and management strengthening in the DHS. The review highlights the increasing attention directed towards leadership and management within health systems in Africa. The majority of WPBL interventions were practice based, with a structured systematic approach to the design and delivery of the intervention, though with considerable diversity in the approaches utilized. Moreover, the review demonstrates the need for focusing on the sustainability and institutionalization of such interventions.

### Synthesis and a modified conceptual framework

**Figure 2.**
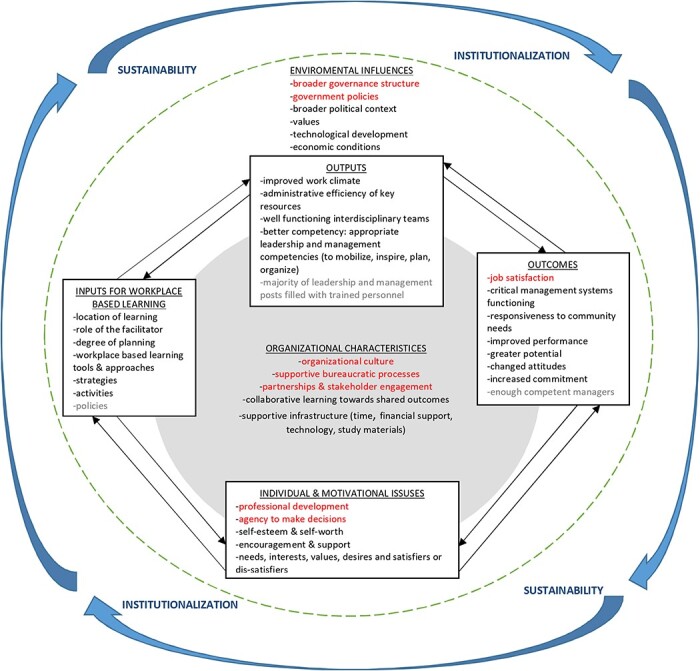
Modified conceptual framework of WPBL (Matthews, 1999; WHO, 2007; Jacobs and Park, 2009; Manley *et al*., 2009; Vriesendorp *et al*., 2010). Greyed out words show concepts from the original framework that were not raised in the papers. Red highlighted words show additional concepts we added. Blue arrows around the framework show sustainability and institutionalization that influence all aspects of WPBL

We employed the adapted framework (Supplementary Appendix 4) to organize and analyse these issues. Based on our findings from this synthesis, we found that the adapted framework does represent a key set of factors associated with WPBL and identifies factors associated with strengthening management and leadership in the public sector. However, we did not find that WPBL interventions resulted in a majority of leadership and management positions being filled with trained personnel, as indicated in Figure 2 (highlighted in grey in the output category). In addition, Matthews (1999) suggests that a key factor for WPBL is the need for policies that facilitate it (highlighted in grey in the input category); however, in this synthesis, we did not find that organizations had such policies. In the review, we did not identify that WPBL interventions resulted in there being enough competent managers within the DHS (highlighted in grey in the outcome category). Although the outcomes in this review do not perfectly align with those depicted in Figure 2, we found that improved health service delivery or health system performance indicated that critical management systems were functioning and managers had become more responsive to community needs through improved service delivery. Additionally, wider organizational learning reflected changes in attitudes, increased commitment and greater potential.

Our synthesis also identified some additional factors (highlighted in red in Figure 2) as important for WPBL and its impact on strengthening management and leadership. We, therefore, made some modifications to the framework to reflect the evidence from the included papers in this study (Figure 2). First, given that the papers in this study did not explicitly apply the adapted framework (Supplementary Appendix 4), we could not easily see a differentiation between individual and motivational issues in the papers. We observed that these factors worked in concert, therefore, for our purposes, we merged the categories of individual and motivational issues as shown in Figure 2. Furthermore, in our papers, we found that the concepts of professional development and agency to make decisions were important factors that seemed to motivate individuals to engage with WPBL. We judge that these concepts add useful specificity to the concepts of needs, interests, desires and satisfiers or dis-satisfiers of learning, as noted in the adapted framework (Supplementary Appendix 4).

Secondly, organizational culture, supportive bureaucratic processes, partnerships and stakeholder engagement were added as organizational characteristics needed to support WPBL. Thirdly, job satisfaction was added as an outcome of WPBL. Fourthly, government policies and the broader governance structure were identified as further environmental determinants of workplace activities. Fifthly, an additional category of sustainability and institutionalization was incorporated. These concepts of sustainability and institutionalization build and further develop the conceptual framework to better understand the longevity of efforts to strengthen district health leadership and management in resource-limited settings through WPBL.

Similar to the initial adapted framework (Supplementary Appendix 4), the eventual modified framework (Figure 2) takes into account internal and external aspects of an organization to highlight that no organization functions in vacuum (Matthews, 1999). Furthermore, the elements of WPBL (inputs, individual and motivational issues, outputs and outcomes) are shown to cut across (Figure 2) the organization and external environment to reflect that organizational and environmental characteristics impact WPBL.

### Lessons for policymakers and those implementing WPBL

Based on the evidence from the included studies, there were numerous ways in which WPBL was successfully carried out. While the WPBL interventions differed in type and nature, as well as length of delivery, the reported studies did not provide conclusive evidence about which approach had a greater influence than others on strengthening district health leadership and management. There were, however, some common lessons about the design and delivery of WPBL interventions that can enable their delivery in the future.

Team-based learning across different layers of the health system promoted distributed and relational leadership and minimized the existing hierarchical structures (Foster *et al*., 2018; Cleary *et al*., 2018a). Peer learning allowed for dialogue among learners where wisdom and knowledge were shared through the interaction of a community of peers (Doherty *et al*., 2018). This enabled health managers to gain confidence in their own ability as leaders through the support they received from others (Foster *et al*., 2018). Reflective practices enhanced critical thinking and nurtured the soft skills needed for interpersonal relationships (Dovey, 2002; Martineau *et al*., 2018; Cleary *et al*., 2018a; Nzinga *et al*., 2021). Though time intensive, action learning and implementing action projects ensured learning was geared towards locally identified organizational needs or gaps (Day, 2001; Matovu *et al*., 2013). It was not only important to support WPBL participants in implementing their learning in the workplace but also to ensure that there were positions available for its graduates (Gormley and McCaffery, 2013; Edwards *et al*., 2015; Johnson *et al*., 2021). This requires engagement and support from employers and senior organizational management (Prashanth *et al*., 2014b; Doherty *et al*., 2018; Chelagat *et al*., 2019; 2021a; Nzinga *et al*., 2021).

In analysing whether WPBL had an impact on strengthening district health leadership and management, the evidence suggested that this depended on the context in which each WPBL intervention was implemented (Doherty *et al*., 2018). All the WPBL interventions encountered organizational and broader socio-political difficulties during implementation. Despite these constraints, WPBL allowed for a much deeper analysis of district problems and led to the development of better strategies to address them (Doherty *et al*., 2018; Martineau *et al*., 2018). DHMTs learnt the importance of selecting strategies that were feasible, effective and affordable given available resources and in-line with other district interventions (Martineau *et al*., 2018). Training key health system managers/leaders on leadership and management was crucial in adopting and implementing principles learnt, as well as improving health system accountability and achieving continued transformation (Matovu *et al*., 2013; Mutale *et al*., 2017; Tomblin Murphy *et al*., 2022). Furthermore, for WPBL to achieve its true potential in impacting district health leadership and management strengthening, it is important to consider its sustainability and institutionalization. The evidence reviewed indicates that sustainability retains new practices in organizations and requires committed funding, flexible design and delivery and that implementing partners have long-term partnerships with health system actors (Sherr *et al*., 2013; Martineau *et al*., 2018; Cleary *et al*., 2018a; Nzinga *et al*., 2021). Institutionalization provides an additional level of consistency and pervasiveness that underscores stability and resilience (Novotná *et al*., 2012; Koon *et al*., 2020). It resulted from ensuring WPBL was embedded in health systems, country owned and offered through national or regional institutions (Mutale *et al*., 2017; Doherty *et al*., 2018; Foster *et al*., 2018; Cleary *et al*., 2018b; Desta *et al*., 2020).

Consistency in learning within health systems is essential, as health systems that fail to learn from their own or others’ experiences risk repeating mistakes. This failure to learn has frequently led to the failure of well-intentioned policies and programmes (Sheikh *et al*., 2020). Therefore, prioritizing learning is critical for strengthening LMIC health systems, ensuring their resilience and equipping them to tackle future challenges more effectively (Sheikh *et al*., 2020, 2022).

## Study limitations

As this synthesis included a heterogeneous set of studies with diverse methodologies that did not apply the framework used in the study, it was not always easy to compare data across publications and fit the data into the precise categories of the framework. Furthermore, we acknowledge that important evidence from other LMIC regions such as Eastern Europe, South America, Central Africa and South East Asia was not identified during the literature search process. This could possibly be due to the poor indexing of research in databases. These studies could provide additional relevant insights on WPBL. We have endeavoured to make the modified WPBL framework to reflect a public sector organizational context, but given the currently limited evidence, further work will be needed to continue to test and refine the framework. Additionally, some of the evaluations of the WPBL interventions were reported by those involved in the funding of the interventions; however, we did not exclude these evaluations.

## Conclusion

This study used a framework approach to synthesize evidence on WPBL for leadership and management development and the impact this learning had on district health leadership and management strengthening. The synthesis reveals that over the last decade, WPBL has received consideration as an approach for leadership and management development. Furthermore, the synthesis provides key lessons for the delivery of WPBL for leadership and management development in the DHS. However, the synthesis is limited by the few papers retrieved, as WPBL is an area of work that is under-studied, particularly in LMICs. The modified WPBL framework can be a useful tool for LMIC policymakers, organizations and health policy and systems researchers to build on to facilitate WPBL in under-resourced settings to strengthen district health leadership and management.

## Supplementary Material

czae095_Supp

## Data Availability

The data generated during the study are available from the corresponding author upon request.

## Footnotes

1. For this review, country-level stakeholders included the national organizations and institutions and/or government agencies within a country that were involved in the interventions, e.g. Ministries of Health (Gilson *et al*., 2012).

2. For this review, external actors included the international individuals, organizations and/or institutions that were involved in the interventions, e.g. the Clinton Foundation HIV/AIDS Initiative (Szlezak *et al*., 2010).

## Abbreviations

AHPSR = Alliance for Health Policy and Systems Research

DHMT(s) = District Health Management Team(s)

DHS = district health system

DIALHS = District Innovation and Action Learning for Health Systems Development

HICs = high-income countries

HIV/AIDS = human immunodeficiency virus/acquired immunodeficiency syndrome

LMICs = low- and middle-income countries

MOH = Ministry of Health

PHC = primary healthcare

SDMT(s) = Sub-District Management Team(s)

UNZA = University of Zambia

WHO = World Health Organization

WPBL = Workplace-based learning

ZMLA = Zambia Management and Leadership Academy

## Reflexivity statement

The authors are all females and represent varying levels of seniority. The corresponding author, based in Kenya, conducted the framework synthesis as part of the requirements for a Master of Public Health degree. While the other authors, experienced health policy and systems researchers based in South Africa, provided supervision for this framework synthesis.
