# Supplementary material for: Workplace-based learning in district health leadership and management strengthening: a framework synthesis
Source: Health Policy Plan. 2024 Oct 9;40(1):105–19. doi: 10.1093/heapol/czae095 (PMC11724643; doi:10.1093/heapol/czae095)
Supplement: czae095_Supp [file czae095_supp.zip › czae095_Supp/Supplementary Appendices.docx]

**Supplementary Appendices**

**Appendix 1.** Summary of literature search strategy

| Key Term (s) or Filters | | Variations | | | | | | | |
| --- | --- | --- | --- | --- | --- | --- | --- | --- | --- |
| work-based learning, workplace learning, workplace based learning, action learning, mentoring, coaching | | "work-based learning" OR "workplace learning" OR "workplace based learning" OR "action learning" OR "mentor s" OR "mentored" OR "mentoring"[MeSH Terms] OR "mentoring" OR "mentors"[MeSH Terms] OR "mentors" OR "mentor" OR "coach" OR "coach s" OR "coached" OR "coaches" OR "mentoring"[MeSH Terms] OR "mentoring" OR "coaching" | | | | | | | |
| leadership, management | | "leadership"[MeSH Terms] OR "leadership” OR "leadership s" OR "leaderships" OR "manage" OR "managed" OR "management s" OR "managements" OR "manager" OR "manager s" OR "managers" OR "manages" OR "managing" OR "management" OR "organization and administration"[MeSH Terms] OR "organization" AND "administration" OR "organization and administration" OR "management" OR "disease management"[MeSH Terms] OR "disease” AND "management" OR "disease management" | | | | | | | |
| health system | | "health system" | | | | | | | |
| LMIC search filters | | “Deprived Country“ OR “Deprived Countries” OR “Deprived Population” OR “Deprived Populations” OR “Developing Countries” OR “Developing Country” OR “Developing Economies” OR “Developing Economy” OR “Developing Nation” OR “Developing Nations” OR “Developing Population” OR “Developing Populations” OR “Developing World” OR “LAMI Countries” OR “LAMI Country” OR “Less Developed Countries” OR “Less Developed Country” OR “Less Developed Economies” OR “Less Developed Nation” OR “Less Developed Nations” OR “Less Developed World” OR “Lesser Developed Countries” OR “Lesser Developed Nations” OR LMIC OR LMICS OR “Low GDP” OR “Low GNP” OR “Low Gross Domestic” OR “Low Gross National” OR “Low Income Countries” OR “Low Income Country” OR “Low Income Economies” OR “Low Income Economy” OR “Low Income Nations” OR “Low Income Population” OR “Low Income Populations” OR “Lower GDP” OR “Lower Gross Domestic” OR “Lower Income Countries” OR “Lower Income Country” OR “Lower Income Nations” OR “Lower Income Population” OR “Lower Income Populations” OR “Middle Income Countries” OR “Middle Income Country” OR “Middle Income Economies” OR “Middle Income Nation” OR “Middle Income Nations” OR “Middle Income Population” OR “Middle Income Populations” OR “Poor Countries” OR “Poor Country” OR “Poor Economies” OR “Poor Economy” OR “Poor Nation” OR “Poor Nations” OR “Poor Population” OR “Poor Populations” OR “Poor World” OR “Poorer Countries” OR “Poorer Economies” OR “Poorer Economy” OR “Poorer Nations” OR “Poorer Population” OR “Poorer Populations” OR “Third World” OR “Transitional Countries” OR “Transitional Country” OR “Transitional Economies” OR “Transitional Economy” OR “Under Developed Countries” OR “Under Developed Country” OR “Under Developed Nations” OR “Under Developed World” OR “Under Served Population” OR “Under Served Populations” OR “Underdeveloped Countries” OR “Underdeveloped Country” OR “Underdeveloped Economies” OR “Underdeveloped Nations” OR “Underdeveloped Population” OR “Underdeveloped World” OR “Underserved Countries” OR “Underserved Nations” OR “Underserved Population” OR “Underserved Populations” OR Afghanistan OR Albania OR Algeria OR “American Samoa” OR Angola OR Armenia OR Azerbaijan OR Bangladesh OR Belarus OR Byelarus OR Belorussia OR Belize OR Benin OR Bhutan OR Bolivia OR Bosnia OR Botswana OR Brazil OR Bulgaria OR Burma OR “Burkina Faso” OR Burundi OR “Cabo Verde” OR “Cape Verde” OR Cambodia OR Cameroon OR “Central African Republic” OR Chad OR China OR Colombia OR Comoros OR Comores OR Comoro OR Congo OR “Costa Rica” OR “Côte d'Ivoire” OR Cuba OR “Democratic People’s Republic of Korea” OR Djibouti OR Dominica OR “Dominican Republic” OR Ecuador OR Egypt OR “El Salvador” OR Eritrea OR Ethiopia OR “Equatorial Guinea” OR Fiji OR Gabon OR Gambia OR Gaza OR “Georgia Republic” OR Georgia OR Ghana OR Grenada OR Grenadines OR Guatemala OR Guinea OR “Guinea Bissau” OR Guyana OR Haiti OR Herzegovina OR Hercegovina OR Honduras OR India OR Indonesia OR Iran OR Iraq OR “Ivory Coast” OR Jamaica OR Jordan OR Kazakhstan OR Kenya OR Kiribati OR Korea OR Kosovo OR Kyrgyz OR Kirghizia OR Kirghiz OR Kyrgyzstan OR “Lao PDR” OR Laos OR Lebanon OR Lesotho OR Liberia OR Libya OR Macedonia OR Madagascar OR Malawi OR Malay OR Malaya OR Malaysia OR Maldives OR Mali OR “Marshall Islands” OR Mauritania OR Mauritius OR Mexico OR Micronesia OR Moldova OR Mongolia OR Montenegro OR Morocco OR Mozambique OR Myanmar OR Namibia OR Nepal OR Nicaragua OR Niger OR Nigeria OR Pakistan OR Palau OR “Papua New Guinea” OR Paraguay OR Peru OR Philippines OR Principe OR Romania OR Rwanda OR Ruanda OR Samoa OR “Sao Tome” OR Senegal OR Serbia OR “Sierra Leone” OR “Solomon Islands” OR Somalia OR “South Africa” OR “South Sudan” OR “Sri Lanka” OR “St Lucia” OR “St Vincent” OR Sudan OR Surinam OR Suriname OR Swaziland OR Syria OR “Syrian Arab Republic” OR Tajikistan OR Tadzhikistan OR Tajikistan OR Tadzhik OR Tanzania OR Thailand OR Timor OR Togo OR Tonga OR Tunisia OR Turkey OR Turkmen OR Turkmenistan OR Tuvalu OR Uganda OR Ukraine OR Uzbek OR Uzbekistan OR Vanuatu OR Venezuela OR Vietnam OR “West Bank” OR Yemen OR Zambia OR Zimbabwe | | | | | | | |
| Publication type | | Journal Article; Full text; Abstract included | | | | | | | |
| Publication date | | 1/1/1990 to 31/5/2024 | | | | | | | |
| Language | | English | | | | | | | |
| Databases | Date of Search | Results | | | | | | | |
|  |  | Total Search | After Removal of Duplicates (n=276) | Articles  Screened | Relevant Articles  Selected  for Title and Abstract Screening | Full-text  Articles  Assessed for Eligibility | Additional Articles from  Purposive search/ Researchers (n=11) | Identified from Citation Tracking/ Reference Lists  (n=4) | Articles Eligible for the Study |
| PubMed | 31/5/24 | 153 |  |  |  | 10 |  |  |  |
| Scopus | 31/5/24 | 145 |  |  |  |  |  |  |  |
| Ebscohost  (Academic Search Premier, AfricaWide Information, CINHAL, HealthSource Nursing, APA PsycInfo) | 31/5/24 | 107 |  |  |  |  |  |  |  |
| Web of Science | 31/5/24 | 172 |  |  |  |  |  |  |  |
| **Total** |  | 577 | 301 | 301 | 22 | 10 | 21 | 25 | 25 |

**Appendix 2.** Summary of articles table

| Author(s) & Year of Publication | Country | Aim of article | Type of WPBL | Stakeholders | Methodology | WPBL Participants &  Setting | Findings |
| --- | --- | --- | --- | --- | --- | --- | --- |
| Chelagat et al. 2019 | Kenya | To investigate the factors influencing knowledge transfer of leadership skills during the implementation of action projects in health care settings, and provide recommendations on strategies or policies to improve knowledge transfer. | Hybrid  9 month LD programme (LeHHO). Strathmore University confers a certificate of achievement on completion. | Driven by country level higher education institution in collaboration with country level actor, and external actor and donor.  (Co-created in 2010 by Strathmore University Business School, MSH and MOH, with funding from USAID for 5 years.) | Qualitative study using in-depth semi-structured interviews. | Participants: 39 FMs from public and private (profit and not-for-profit) health facilities    Setting: 19 counties  Study covered a period of 7 years | Timely completion of action projects was dependent on: context specific need-based training; workplace environment; trainee’s motivation and leadership positions; team-based coaching; and the ability to leverage contextual opportunities. Barriers to the transfer of learned leadership knowledge to the workplace included: poor management support; insufficient team or staff support; high staff turnover; misalignment of priorities between managers and senior leadership; lack of technical capacity needed to implement projects; endemic strikes (public sector facilities); lack of political goodwill and/or political interference; and poor communication among key stakeholders. To improve leadership knowledge transfer, programme alumni proposed: effective financial and human resource allocation and utilization; action projects to be in alignment with organizational priorities; effective communication among key stakeholders to ensure ample buy-in; workplace teams to undergo leadership training together to ensure sustainability of institutional improvements. |
| Chelagat et al. 2020 | Kenya | To evaluate the impact of project-based experiential learning on health service delivery. | Hybrid  9 month LD programme (LeHHO). Strathmore University confers a certificate of achievement on completion. | Driven by country level higher education institution in collaboration with country level actor, and external actor and donor.  (Co-created in 2010 by Strathmore University Business School, MSH and MOH, with funding from USAID for 5 years.) | Quasi-experimental time-series study with non-random sampling using quantitative data (close-ended questionnaires and HMIS service delivery indicators data) and qualitative data (Challenge Model documents and programme reports) | Participants: 15 FMs from public and private (profit and not-for-profit) facilities  Setting: 13 counties (Nairobi, Elgeyo Marakwet, Kisumu, Samburu, Nakuru, Busia, Kisii, Siaya, Uasin-Gishu, Kakamega, Kajiado, Kiambu and Mandera)  Study covered a period of 7 years | Participation in the LD programme contributed to a significant increase in health service delivery through achieving action project goals. The action projects generated immediate application of knowledge learned to the workplace. This was observed across different health sectors both public and private. Additionally, improved health service indicators were sustained for 60 months after training. Participants attributed the success of the action projects to the team-based coaching sessions built around institutional priorities. |
| Chelagat et al. 2021 | Kenya | To assess the effectiveness and impact of LD training on health system performance during the implementation of action projects. | Hybrid  9 month LD programme (LeHHO). Strathmore University confers a certificate of achievement on completion. | Driven by country level higher education institution in collaboration with country level actor, and external actor and donor.  (Co-created in 2010 by Strathmore University Business School, MSH and MOH, with funding from USAID for 5 years.) | Quasi-experimental time-series study with non-random sampling using quantitative data (close-ended questionnaires) and qualitative data (Challenge Model documents and in-depth interview guide) | Participants: 31 health managers from public and private (profit and not-for-profit) institutions  Study covered a period of 6 years | The LD training had a positive impact on health system performance and efficiency indicators as reflected by the action projects. Training (compared to no training) had positive impacts on the health system pillars of service delivery, HIS, leadership and governance, health workforce, financing, and access to essential medicines. Projects focusing on HRH were the least successfully implemented. Some projects were unsustainable due to team members leaving institutions. |
| Chelagat et al. 2021 | Kenya | To investigate the drivers and inhibitors of sustainability of action projects; to inform Kenya’s healthcare sector on strategies or policies for sustainability of project results. | Hybrid  9 month LD programme (LeHHO). Strathmore University confers a certificate of achievement on completion. | Driven by country level higher education institution in collaboration with country level actor, and external actor and donor.  (Co-created in 2010 by Strathmore University Business School, MSH and MOH, with funding from USAID for 5 years.) | Qualitative study using in-depth semi-structured interviews | Participants: 33 managers from public and private (profit and not-for-profit) health facilities  Setting: 19 counties  Study covered a period of 6 years | 33 out of 39 action projects were successfully sustained 2-5 years after the LD training. Key drivers of sustainability were: programme design (workplace team recruitment, use of the Challenge Model and team coaching); stakeholder buy-in; improved communication skills; inclusion of senior organizational leadership; continued presence of the change champion (trained manager); and devolution and political good-will. Inhibitors of sustainability included: inadequate HRH (public and faith-based facilities); misalignment of priorities between managers and senior leadership; managers’ workloads (most were practicing clinicians); and devolution and political interference. |
| Choonara et al.  2017 | South Africa | To demonstrate the importance of informal learning strategies in nurturing the development of leadership skills. | Practice based  (Not an explicit WPBL intervention; however WPBL was observed and these findings documented while the researcher was conducting research on district financial management.) | Driven by country level higher education and country level research institution.  (University of the Witwatersrand – Centre for Health Policy, MRC Health Policy Research Group.) | Qualitative case study design using in-depth interviews and non-participant and participant observations | Participants: 18 interdisciplinary (finance, HR, IT, HIS and procurement) district and sub-district participants involved in district financial management  Setting: ‘Isikhala’ district in Gauteng Province | Despite a decentralized health system, decision-making assigned in theory but not in practice. Structural constraints such as centralization of bureaucratic processes led to: difficulty in accessing financial resources; delayed procurement; lack of IT equipment and slow internet connectivity. Regardless of these constraints, on the job learning was observed (learning from others, communication, delegation between staff and team-based learning) which developed a group of leaders able to problem solve and motivate staff. |
| Cleary et al. 2018 | South Africa | To explore whether and how an action learning LD intervention has enabled relational leadership in a resource limited setting. | Practice based  5 years of WPBL embedded in the broader DIALHS action-research project. | Driven by country level actors and country level higher education institution.  (Collaboration between the Provincial Government of the Western Cape health department, City of Cape Town health department, School of Public Health University of the Western Cape, and School of Public Health and Family Medicine UCT.) | Qualitative evaluation study using document reviews, in-depth interviews,  group discussions, observations of practice and of LD interventions,  and notes from reflection sessions | Participants: FMs from 9 PHC clinics and 6 members of the SDMT  Setting: Mitchell’s Plain sub-district within  the City of Cape Town in the Western Cape Province | Group-based LD interventions across two different layers of the health system promoted distributed and relational leadership. But the broader hierarchical governance context with its central accountability processes limited the impact of relational LD within the sub-district. |
| Desta et al. 2020 | Ethiopia | To assess the capacities and performances of districts receiving training in leadership, management and governance compared to the districts that did not receiving the training. | Practice based  WPBL embedded in the 5 year USAID Transform Primary Health Care Project | Driven by external actor(s) and donor  Collaboration of MOH, project consortium (Pathfinder International, JSI Research & Training Institute Inc., Abt Associates, EnCompass LLC, and the Ethiopian Midwives Association), and local NGOs, with funding from USAID | Quantitative cross  sectional study using USAID Transform Primary Health Care supervision  questionnaires over a period of 1 year | Participants: district managers and staff from PHC facilities  Setting: 284 districts  Study covered 1 fiscal year (2019) | The leadership, management and governance training taught critical skills of leadership and management together with problem solving skills through the management improvement projects and coaching. This brought about: improved district management practices and structure; district capacity; and quality of care. |
| Doherty et al. 2018 | South Africa | To assess the health leadership training programme at UCT between the period of 2008 and 2014, to generate lessons that inform others involved in LD programmes in LMICs, on the techniques used, challenges faced, and what influences programme success. | Hybrid  18 month health leadership training programme. WPBL embedded as part of its format. UCT confers a PG Dip. on completion. | Driven by country level higher education institution.  (Division of Health Policy and Systems, School of Public Health and Family Medicine, UCT.) | Descriptive study using mixed methods techniques which included a document review of programme materials, brief alumni survey questionnaires, and semi-structured telephonic interviews | Participants: District and Facility Managers (91 participants from across South Africa over a period of 6 years) | Managers’ workloads proved to be a constraint to some completing the programme. Difficult workplace dynamics due to power imbalances limited action learning processes. Workplace line managers were not always cooperative to students’ study needs between modules. Poor public sector leadership capacity and poor human resource development processes also limited monitoring the impact of the programme among trainees. Resource constraints make it difficult to gain a critical mass of well-trained public sector leaders to bring about change in organizational culture. |
| Dovey 2002 | South Africa | To describe the outcomes of a module of WPBL in developing leadership skills. | Hybrid  2 year district health leadership course. WPBL embedded as part of its format. Rhodes University confers a Certificate in District Health Management on completion. | Driven by country level higher education institution, in collaboration with country level actors, and external actors and donors.  (Collaboration of grassroots community organizations, Rhodes University J&J Leadership Development Institute, MSH contracted by USAID, and Eastern Cape Department of Health) | Case study (no description on how data was collected) | Participants: DHMTs (12-20 per team)  Setting: Eastern Cape Province | Committed coaching (by Rhodes staff) was a key factor in developing DHMTs capacity to successfully manage the politics of implementation of work-based projects. The focus of the programme was on the nature of learning gained and not whether the work-based projects succeeded or failed. Teams completing projects demonstrate a sustained culture of collaborative action, with the development of strategic thinking capacity and power management strategies. Poor provincial leadership put DHMTs capacity gains at risk. |
| Edwards et al. 2015 | Mozambique | To evaluate the impact of a health management mentorship programme on health system strengthening. | Practice based  Pilot done over 1 year (2013) | Driven by external actor  (FGH, Vanderbilt Institute for Global Health, MOH, CDC and funded by PEPFAR) | Quantitative study using metrics of 63 Indicators of health system strength focusing on accounting, human resources, monitoring and evaluation capacity, and transportation management over a period of 1year | Participants: District health managers and administrators  Setting: 10 districts in Zambézia province | The site-based health management mentoring intervention led to: improvements in accounting; human resources (though not in the completion of staff performance evaluations); monitoring and evaluation capacity (specifically in improvement in stock availability of administrative forms); and transportation management. Accounting displayed the greatest and most sustained improvements. Despite a limited health workforce, receiving salaries on time and opportunities for professional development proved crucial for health worker motivation and retention. Challenges to the programme included: constant changes to donor and MOH priorities; an incomplete decentralization process that led to unpredictable decision making; an increasing demand for health services adding an extra strain to ensure quality; and different planning and fiscal years between MOH and FGH (like staff away for mandatory national trainings and meetings.) |
| Foster et al. 2018 | Zambia | To describe the design, development and outcomes of a LD programme for nurse managers in a low-resource setting at the subnational level and the key factors for improving programme success. | Practice based  12 month leadership and management programme for nurses. WPBL embedded as part of its format. UNZA confers a Certificate in Leadership and Management Practice on completion. | Driven by country level actor in collaboration with country level higher education institution, and external actors/donors.  (Collaboration of MOH, UNZA, IntraHealth International, J&J, mPowering Frontline Health Workers, ZUNO, General Nursing Council and Health Professionals Council of Zambia) | Mixed-methods study using evaluation of health project presentations, interviews with key stakeholders (nurses, clinical officers, environmental health technicians and CHAs.) FGDs with neighbourhood health committee members and community volunteers | Participants: 18 nurse facility heads and 5 district nurse supervisors  Setting: 18 rural facilities across 5 districts. | Joint participation of facility heads and district managers improved their relationships and the oversight and accountability for community health. Facility heads strengthened their leadership and management skills with proven progress in the quality and accessibility of health services. They increased their ability to lead frontline teams as well as enhance their skills and confidence in using technology. Key factors for programme success include: country ownership; community engagement; key stakeholder participation; and making the best use of resources to ensure programme remained financial viable. |
| Gormley and McCaffery 2013 | Uganda | To describe the factors that lead to a successful LD programme for HRH managers at the district level and offer lessons that can be used in other countries for HRH development programmes at regional levels. | Practice based  6 month short course; part of the CapacityPlus Project (USAID’s global project dedicated to health workforce.) | Collaboration of country level actors and external actor and donor  (Collaboration between MOH, USAID, and IntraHealth International) | Desk review of relevant programme materials, interviews with key stakeholders (district health leaders, programme designers, managers and instructors.) FGDs with HRH teams who went through the programme | Participants: District HRH teams (63 participants)  Setting: 14 districts | Change in individual and organizational performance requires supportive organizational changes. Effective district HRH development programmes need: key stakeholders that are involved in the design and delivery of the programme; learning delivered through practical, participant-focused adult learning methods; individuals selected based on HRH work responsibilities and attended as a team; on-the-job coaching to assist in implementing changes in the work environment; and quality maintained throughout the programme. HRH development programmes need to align with national and sub-national goals. |
| Kwamie et al. 2014 | Ghana | To explore the results, contexts, and tools of a management and leadership intervention for district health managers and whether the intervention supported systems thinking among the managers. | Practice based  6 month (February to August 2012) once off course. (No new LD programme cycle afterwards and no funds set aside for it.) | Driven by country level actor.  (Developed by MSH and implemented by the GHS) | Realist evaluation using document reviews (team meetings’ minutes, training workshop materials, teams’ presentations and action plans, reports from previous  LD programme cycles, and national, regional and district policies), participant observations (district and regional health team meetings, workshops, LD programme activities, coaching sessions, and day-to-day district activities) and semi-structured interviews (district managers and programme facilitators) | Participants: 5 teams (4-7 members) of District managers (the DHMT; 3 SDMTs and members of the district hospital management team)  Setting: Rural district of Dangme West | Initially the novelty of the leadership development programme was received with positivity as district managers did not have formalized leadership or management training. But implementation of the programme was resource intensive and required staff to leave work to attend trainings. In addition the top-down manner in which the leadership programme was introduced reinforced the hierarchical, highly centralized, decision-making processes within the health system and this compromised institutionalization of the programme among district managers. As a result, district manager decision space remained narrow. |
| Mansour et al. 2010 | Egypt | To describe the outcomes of a LD programme for health workers to improve service delivery challenges and explore the success in scaling up the programme. | Practice based  Several Months (not specific in article). Took 4 years to cover all of Aswan Governorate. Initially a 1 year donor funded pilot by USAID. Then taken over by a local team for scale up. | Driven by country level actor.  (Initially funded by USAID and co-led by MOH and MSH. Then taken over by a local Aswan Governorate team for scale up) | None given. More of a report of the LD Programme scale-up | Participants: 1000 health workers (in teams) from 184 PHC facilities and governorate managers  Setting: Rural and mostly impoverished Aswan Governorate | Through applying leadership and management practices, teams learned to work together and mobilize other stakeholders to address public health problems. Through increased commitment and ownership of problems, teams were able to scale up the LD programme using local resources, once donor funded ended. Some challenges of the LD programme included: advocating its benefit to higher MOH management; finding additional facilitators when core facilitators got busy; and addressing health worker turnover during programme. Due to sustainability and scale-up in Aswan, the LD programme has been implemented in other governorates in Egypt and in 35 countries in the global South. |
| Martineau et al. 2018 | Ghana,  Tanzania and Uganda | To explore ways to strengthen decentralized health management to improve health worker performance in sub-Saharan Africa. | Practice based  2 years of WPBL embedded within the PERFORM action research project. | Driven by country level higher education institutions, in collaboration with external higher education institutions and donor.  [(The PERFORM consortium: Liverpool School of Tropical Medicine, University of Leeds, University of Ghana, Swiss Tropical and Public Health Institute, Institute of Development Studies, University of Dar-es-Salaam, School of Public Health, Makerere University), and funded by the European  Commission’s Seventh Framework programme] | Qualitative Evaluation study using a document review (workshop reports, work plans and progress reports, reflective diaries and follow-up visit reports), 50 in-depth interviews and 6 FGDs | Participants: DHMTs  Setting: 9 districts (mix of rural, urban or semi-urban) across 3 countries (3 districts from each country) | The root cause of health worker performance problems was due to poor human resource management systems. Going through the programme allowed for a much deeper analysis of district problems and therefore better strategies to address them. DHMTs learnt the importance of selecting strategies which were feasible, effective, and affordable given available resources; and in-line with other district interventions. Funding for action learning projects needs to be addressed to ensure suitability and sustainability of the programme. Several intervention cycles in the same DHMTs are needed to begin to see significant impact on organizational culture. As such stakeholder expectations need to be managed regarding outcomes. |
| Mutale et al. 2017 | Zambia | To evaluate the impact of a leadership and management training programme as a facilitator to health system strengthening in resource limited settings | Practice based  6-12 months in-service leadership and management course called ZMLA | Driven by country level actor  (Course development supported by MOH, Ministry of Community Development, Mother and Child Health, BroadReach Institute for Training and Education and the Zambia Integrated Health Systems Strengthening Project) | Cross-sectional mixed method study using 444 trainee survey questionnaires, 70 key informant interviews (trainees, course implementers, mentors, selected stakeholders, and provincial, district and health facility managers), trainee knowledge quizzes, trainee feedback forms, and trainee case studies | Participants: 767 health workers (health system managers who came as teams from the same district or facility)  Setting: Designated districts across Zambia covering a period of 4 years | Training key institutional health system managers/leaders was crucial in adopting and implementing principles learnt, as well as improving health system accountability. Certification by NIPA and obtaining diplomas made the training very popular among busy health workers. Many trainees did not implement the solutions generated to solve workplace problems as graduation did not depend on this. Questions of sustainability arose as the programme was a free donor funded course. Offering it through a local institution could mitigate this issue. |
| Nakanjako et al. 2015 | Uganda | To describe lessons learned from an experiential leadership training programme to guide further leadership development programmes for nurses and doctors in LMICs | Hybrid  1 year Afya Bora Global Health Leadership Fellowship (4 fellows per year) | Driven by country level higher education  (Makerere University, College of Health Sciences, MOH, PEPFAR and NGOs - not specified which ones) | Document review of weekly mentor-fellow meetings and monthly team meeting reports | Participants: 15 healthcare workers (nurses and doctors)  Study covered a period of 4 years | The programme demonstrated that it is possible to strengthen individual and organizational capacity (to address health system challenges) through WPBL. Key to the success of the programme was: matching fellows to projects that were equally valuable to both the fellow and organization; mentorship; and allocating protected time for fellows to address problems. To ensure sustainability: the programme was co-led by both US and Ugandan trainers; and programme alumni were invited to take part in future programme activities. |
| Nzinga et al. 2021 | Kenya | To explore the design and development of an innovative LD intervention, to provide insights and lessons to other LMIC health settings, on how leadership skills can support in building resilient health system actors. | Practice based  9 months of WPBL embedded in broader governance research of the RESYST Consortium in Kilifi county | Driven by country level research institution  (Health policy and systems researchers of the RESYST Consortium in Kenya and health system managers of Kilifi County) | Qualitative study using participant observations of meetings and managers’ interactions, 9 in-depth interviews with participating managers, document reviews (meeting notes, participant diaries and reflective assignments, pre- and post-activity evaluations, and workshop planning and meeting notes) | Participants: 30 sub-county health managers and FMs (in teams)  Setting: Kilifi County | WPBL created safe spaces for team sharing and reflection, and nurtured the soft skills needed for interpersonal relationships. Managers gained a greater appreciation for health system software, improved their self-awareness and communication skills, and role-modelled positive behaviour to teams. Collaborative decision-making helped strengthen teams. New skills led to job satisfaction and increased work commitment. Managers who had supportive networks and passionate about their roles were able to consistently attend the training. To sustain new skills and see transformation in organizational climate, managers stated more time was needed to implement changes as well as the need for senior management involvement and support. Hindrances to seeing sustained improvement in the health system included: hierarchical governance structure within the health system; political interference; distrust among health system actors and the need to balance managers’ heavy workloads. |
| Prashanth et al. 2014 (2) | India | To explore how a capacity building intervention for health managers evolved over time and how the intervention translates into improved performance of managers’ planning and supervision tasks. | Hybrid  30 month capacity-building intervention  (5 days for medical officers and a single discussion day for PRI representatives) | Driven by country level actors  [Swasthya Karnataka (a consortium of five NGOs: Institute of Public Health, Bangalore; Centre for Global Health Research, Bangalore; Centre for Leadership and Management in Public Services, Bangalore; Institute of Health Management and Research, Bangalore; and Karuna Trust) and the Karnataka state government] | Realist evaluation using qualitative data (27 in-depth interviews, participant observations of monthly and annual district review meetings, government documents, and relevant training materials, reports  notes), and quantitative data (surveys, questionnaires, and annual district data reports) | Participants: Teams of health managers, 162 PHC medical officers, and PRI representatives  Setting: Tumkur district in the Karnataka state of southern India | Managers who fully participated in the training and expressed intentions to make changes in the workplace did not always realize those changes. This was often due to the context of the sub-system in which proposed changes were to be implemented in. These sub-system contexts determined how the same intervention worked in one setting but not in another. Hindrances included: poorer resourced parts of the district; increasing interference from elected local government officials; and lack of senior management support to improve health system performance. However improved organizational performance could be achieved by decreasing the power gap between doctors and the rest of the health management team, and cultivating teamwork among interdisciplinary team members. |
| Rowe et al. 2010 | Liberia | To describe the transfer of a health management programme from a north to south institution and offer key factors needed for programme sustainability, scale up and replication. | Hybrid  6 month short course | Driven by both country level and external higher education institution  (Collaboration between Mother Patern College of Health Sciences, Stella Maris Polytechnic, Yale University School of Public Health, and CHAI) | Quantitative study using self-administered participant surveys | Participants: 93 managers from County Health Teams, MOH, health facilities and NGOs (2-3 members from each institution attended)  Setting: All 15 counties of the country  Study covered a period of about 1 and ½ years (2017-2019) | The programme demonstrated a successful transfer of a training programme from a northern to southern institution. Participants continually reported a significant improvement in management skills during the transfer of the programme. Key elements for an effective management training programme include: use of a short course format focusing on a few vital skill areas with practical tools for application; including a mix of classroom modules, action projects, and on-site mentoring; collaborating with key stakeholders (like MOH and Mother Patern College) to enable sustainability and scalability; and ensuring facilitators/mentors are well trained. |
| Seims et al. 2012 | Kenya | To assess if strengthening leadership and management skills of district and facility health teams results in increased health service delivery and coverage. | Practice based  6 months short course | Not really explicitly stated (except MOH) but from authors information – MSH, MOH and USAID | Non-randomized quasi-experimental study with comparison groups using quantitative data of health service indicators from the Kenyan HMIS, and qualitative data from either in-person or telephonic interviews | Participants: 67 teams of DHMTs and FMs  Setting: from 6 provinces (Rift Valley, Nyanza, Central, Nairobi, Eastern and North Eastern)  Study covered a period of 3 years | Strengthening team-based leadership and management contributes to improvements in health service delivery and these positive outcomes can be sustained for 6 months after the LD programme. Significant increases were seen in: district health service coverage of children under 1 year vaccinations; skilled birth attendant deliveries; and number of facility antenatal visits. Staff, medicine and vaccine shortages, and drought and insecurity were the key factors that led to some teams being unable to sustain improvements. |
| Sherr et al. 2013 | Mozambique | To assess the impact of building district management capacity through data-driven decision making on under-5 mortality. | Practice based  WPBL embedded in the 7 year Mozambique PHIT partnership project | Driven by external actor  [PHIT partners/ consortium (Sofala Provincial Health Directorate, MOH Beira Operations Research Center, HAI, University of Washington Global Health and Industrial and Systems Engineering departments, and Eduardo Mondlane University School of Medicine)] | Quasi-experimental controlled time-series study using quantitative data (national demographic and health surveys, national multi-indicator cluster surveys, patient satisfaction questionnaires, and facility and district surveys) and qualitative data (participant observations) | Participants: FMs from 146 facilities and district managers  Setting: All 13 districts in Sofala Province | It was difficult to define the role and thus strengthen the capacity of district managers due to the slow and uneven decentralization process. The extreme human resource constraint made it difficult to have personnel solely take on management positions. Despite these challenges, adaptations were made to the management and leadership training that led to more of a focus on mentoring participants towards implementing data-driven decision making. |
| Tetui et al. 2017 | Uganda | To explore the impacts of a participatory action research approach to strengthen health managers’ capacity in LMICs | Practice based  4 years of WPBL embedded in the participatory action research of the MANIFEST Project to improve maternal and neonatal health outcomes. The PAR cycle was repeated 4 times a year refining issues or tackling new ones. | Driven by country level higher education institution.  School of Public Health, Makerere University | Qualitative study using 16 key informant interviews, 18 document reviews of meetings’ minutes, and participant observations of project activities and meetings | Participants: 42 health managers consisting of district level managers and FMs  Setting: Kamuli,  Pallisa and Kibuku districts of eastern Uganda | Initially the desire to maintain the status quo was at odds with the need for innovation and change. With improved trust among stakeholders, partnership and commitment towards new ideas, was initiated and sustained. The participatory action research approach: improved managers’ capacity to collaborate with different stakeholders; nurtured creativity in meeting community needs; and provided skills in planning, coordinating and achieving goals in the midst of different levels of authority and power within the health system. Additionally the managers developed analytical reflection skills and a process review culture. Despite these improvements there was still a dependency on the WPBL external implementing partners for project coordination supervision. |
| Tomblin Murphy et al. 2022 | Tanzania | To evaluate the effectiveness of a leadership and managerial capacity building initiative aimed at improving the quality of MNH in PHC facilities. | Hybrid  3 years (2018-2021)  of WPBL embedded within the ASDIT project. | Not really explicitly stated but from authors information collaboration between country level higher education institutions, external higher education institutions and external actors and donors.  (Muhimbili University  for Health and Allied Sciences, St Francis University College of Health and Allied  Sciences, Tanzanian Training Centre for International  Health, Dalhousie University WHO/PAHO Collaborating  Centre on Health Workforce Planning, Nova Scotia Health, and IWK Health Centre) co-funded by the CIHR and  IDRC | Mixed-methods study using quantitative data  (survey questionnaires and star-rating assessments) and qualitative data (FGDs, and informal  discussions with stakeholders during mentoring visits  and regularly CHMTs and FMs meetings.) | Participants: 30 (health providers and managers from 20 PHC facilities, 8 members from 4 CHMTs from Gairo, Morogoro, Kilosa and Mvomero district councils, and 1 member from the RHMT.)  Setting: Morogoro region  The primary ASDIT study covered a period of 6 years (2015-2021) | Leadership and managerial skills in participating health facilities improved, enhancing communication between health facility staff, FMs, CHMTs and the RHMT. This created a more supportive workplace environment, leading to enhanced teamwork, job satisfaction, productivity, and improved services for mothers and new-borns. Star-ratings, measuring overall facility leadership and management capability, increased in 15 out of 19 facilities, with the number of facilities achieving the target of 3 plus stars rising from 2 in 2018 to 10 in 2021. Improvements in team climate, role clarity/conflict, and job satisfaction among health providers and managers was also observed, highlighting the positive impact on workplace environment and service quality for MNH. |
| **Key:** ASDIT – Accessing Safe Deliveries in Tanzania; CDC – United States Centers for Disease Control and Prevention; CHAs – Community Health Assistants; CHAI – Clinton Foundation HIV/AIDS Initiative; CHMTs – Council Health Management Teams; CIHR – Canadian Institutes of Health Research; DHMTs – District Health Management Teams; DIALHS – District Innovation and Action Learning for Health Systems Development; FGDs – Focus Group Discussions; FGH - Friends in Global Health; FMs – facility managers; GHS – Ghana Health Services; HAI – Health Alliance International; HIS – health information systems; HMIS – Health Management Information System; HR – human resources; HRH – Human Resources for Health; IDRC – Canada’s International Development Research Centre; IT – information technology; J&J – Johnson and Johnson; LD – leadership development; LeHHO – Leading High-performing Healthcare Organizations; LMICs – Low-and Middle-Income Countries; MANIFEST – Maternal and Neonatal Implementation for Equitable Systems; MOH – Ministry of Health; MNH – Maternal and New-born Health; MRC – Medical Research Group; MSH – Management Sciences for Health; NGOs – Non-governmental organisations; NIPA – National Institute for Public Administration; PAR – Participatory Action Research; PEPFAR – President's Emergency Plan for AIDS Relief; PG Dip – Postgraduate Diploma; PHC – primary healthcare; PHIT – Population Health Implementation and Training; PRI – Panchayati Raj Institution (elected members of local government); RESYST – Resilient and Responsive Health Systems; RHMT – Regional Health Management Team; SDMT – Sub-District Management Team; UCT – University of Cape Town; US – United States; USAID – United States Agency for International Development; UNZA – University of Zambia; WPBL – Workplace based learning; ZMLA – Zambia Management and Leadership Academy; ZUNO – Zambian Union of Nurses Organization; WHO/PAHO – World Health Organization/Pan American Health Organization | | | | | | | |

**Appendix 3.** Data extraction table

| Author(s) & Year of Publication | Inputs for WPBL | Organizational  Characteristics | Individual & Motivational Issues | Environment  Influences | Outputs | Outcomes | Sustainability/ Institutionalization |
| --- | --- | --- | --- | --- | --- | --- | --- |
| Chelagat et al. 2019, 2020,  2021 (2) | - 4 team coaching sessions (1-2 hours each) - 5 workshop modules (4 classroom days each) - 1 cross-learning site visit - Peer/group learning - Flipped classrooms using cases studies - Systematic approach to leadership training (training structured around MSH’s integrated Leadership Management and Governance results Framework) - Action Project (institutional improvement project using MSH’s Challenge Model) | Challenges   - Poor improvement in health indicators and health system performance post-devolution - In public institutions - prevalent health worker strikes - poor working conditions - staff shortage - low salaries - limited resources - In faith-based and public institution - high staff turnover - poor staff retention - lack of job security - scarce medical supplies   Opportunities   - Power and position - Those participants in senior positions were able to expedite implementation of action projects | Motivation directly linked to WPBL   - Participants increased in their self-awareness - Participants highly appreciated team coaching - Coaches motivated managers to achieve project goals | Challenges   - Devolution reforms challenging to implement   Opportunities   - Health management and service delivery transferred to 47 semi-autonomous counties - Political goodwill - Facilities with county government political support had action projects implemented on time | Positive   - Participants learnt to communicate effectively and listen to team members - 93% of action projects attained desirable results - Teamwork was cultivated and team meetings promoted shared vision - Positive work climate throughout the action project | Positive   - Improvement in health service delivery - 85% of action projects were sustained for a period of 2–5 years after LD training - Managers’ attitudes changed to being more positive - Positive impact on health system performance and efficiency indicators: increase in skilled birth attendance; full child immunizations; utilization of in- and out-patient services; patient satisfaction; and reduced out-patient turnaround time   Negative   - Teams prioritized action projects within their sphere of influence and control. As such HRH and health financing challenges were rarely selected (despite HRH being a major challenge in both public and faith-based facilities) | - Implementation of action projects during and after the LD programme depended upon on how well participants could creatively use existing resources to produce positive change within their institutions - Key drivers of sustainability were: programme design (workplace team recruitment, use of the Challenge Model and team coaching); stakeholder buy-in; improved communication skills; inclusion of senior organizational leadership; continued presence of the change champion (trained manager); and devolution and political good-will. - Inhibitors of sustainability included: lack of technical capacity needed to implement projects; endemic strikes (public sector facilities); misalignment of priorities between managers and senior leadership; poor management support; managers’ workloads (most were practicing clinicians); devolution; lack of political goodwill and/or political interference; and poor communication among key stakeholders |
| Choonara et al.  2017 | - Learning from other leaders/superiors through communication and delegation - Team-based learning - Peer-learning – staff worked together on different tasks - Each staff member trained on every finance-related task | Challenges   - Lack of agency prevented district staff from implementing solutions for problems - Structural constraints - centralization of bureaucratic processes leading to: difficulty in accessing financial resources; delayed procurement; lack of IT equipment and slow internet connectivity - inadequate delegation or authority - lack of administrative capacity at sub-district levels   Opportunities   - Positive learning environment - District manager put emphasis on having good relationships with staff (through communication) | Motivation directly linked to WPBL   - Delegation of tasks to lower level staff motivated and empowered them, and reduced managers’ workloads - Good work was acknowledged - District annual awards for hard work, punctuality and innovation - Continuous learning environment | Challenges   - Despite decentralized health system, decision-making assigned in theory but not in practice - Budgets and requests had to be approved by Provincial Treasury Department (not the Provincial Department of Health) - Lose of institutional memory (with exodus of healthcare workers) post 1994 | Positive   - Improved teamwork in the finance department - Participants able to express agency when dealing with complex district constraints - Participants able to analyse and resolve existing and anticipated problems - Participants able to manage and empower staff - Participants able to inspire staff to achieve district goals | Positive   - Participants able to generate and share new knowledge and learning to enhance collective knowledge within the district - Participants developed agency to address DHS constraints - Broader district staff became solution-driven and motivated   Negative   - At the sub-district level (due to structural constraints and a lack of administrative capacity), it was more difficult to be creative and innovative at solving health system challenges. | (Not an explicit WPBL intervention, however WPBL was observed and these findings documented while the researcher was conducting research on district financial management) |
| Cleary et al. 2018 | - Action learning - Group reflection practices - Group coaching - 5-day health management short course - Several day-long workshops - Peer support meetings - Active facilitation of relational leadership workshop; reflection sessions; peer support meetings and coaching sessions - Structured programme planning (around the “Thinking Environment” principles) | Challenges  Centralized accountability processes. Lengthy bureaucratic mechanisms to ensure accountability to city, provincial and national levels; as such support and mentoring of staff driven by a compliance-focused approach i.e. a detailed audit report.  Opportunities  Willingness by the sub-district to engage in what would be considered a very non-traditional form of work placed based learning in the public sector. | General motivation in the workplace   - FMs feel victims of the system - FMs not always trusted by management to make decisions based on their local context - Accountability mechanisms demotivating to FMs   Motivation directly linked to WPBL   - Verbalized appreciation was highly valued and motivated managers |  | Positive   - Better trust and cohesion among the SDMT as well as within relationships between FMs and staff - FMs given more discretionary   decision space   - Improvements in engagements with accountability processes and supervisory meetings | Positive   - Shift in the organizational culture whereby FMs became more engaged and assertive especially in meetings and expressed concern if new initiatives were unrealistic to implement - SDMT and FMs understood benefits of relational leadership   Negative   - Hierarchical governance structure persisted as senior/higher level managers did not undergone the LD intervention | - Prolonged engagement of LD implementing team/researchers with participants. (The LD intervention emerged through several engagements over a period of time between the research team and district/facility managers.) |
| Desta et al. 2020 | - Leadership, management and governance trainings - Health service management improvement project linked to MCH - On-site coaching - Learning sessions during performance review meetings |  |  | Opportunities   - MOH introduced in-service training on Leadership, Management and Governance in 2017 | Positive   - Improved management practices and structure - Teams strengthened - Improved availability of functional equipment - Improved availability of health workers | Positive   - Improved quality of services at facility level - Established multisector collaboration | - USAID funded - Project supports government initiatives (leadership, management and governance trainings) and thus integrates LD as part of its activities. |
| Doherty et al. 2018 | - Mentorship - Networking - Reflective practice - 4 (5-8 day) classroom modules - Health system intervention project - Active facilitation (of classroom modules) - Structured programme planning (to ensure learning objectives were known by participants) - Peer-learning with discussions of case studies - Problem-based learning - Practice-linked assignments geared towards workplace challenges (action learning) - Diploma in Health Management | Challenges   - Managers’ workloads proved to be a constraint to some completing the programme - Workplace line managers not always cooperative to students study needs between modules - Heavy workloads made networking and mentorship difficult to achieve - Misaligned expectations between work-based mentors and students - Lack of active support at work for students to practice skills acquired nor flexibility to make mistakes - Mismatched students selected for the programme - Budget constraints - Staff shortage - Poor senior leadership capacity - Silo mentality among staff in different departments - Centralized bureaucratic authority and processes - Lack of a culture of excellence - Resistance to change from mangers and staff who had not undergone the training   Opportunities   - Willingness by the city, provincial and national departments of health to nominate students to the programme | Motivation directly linked to WPBL   - Enthusiasm for the programme - Satisfaction with structure, content and teaching style of programme   including the workplace strengthening   - unleashed potential as participants realized that their characteristics and   experiences were worthwhile; and change was possible | Challenges   - Political interference | Positive   - Managers improved their presentation skills for meetings - Managers able to identify and analyse root causes of problems in the workplace, and strategize on how to improve - Managers able to engage positively with staff and stakeholders - Managers understood their roles and personal strengths and weaknesses,   thus able to transform interpersonal relationships with staff, supervisors and stakeholders   - Managers learnt to communicate more effectively and involve others in decision-making | Positive   - An alumni network for on-going support - Developed high level and good quality managers in the public health sector - Managers remained in the public health sector and in the same province - Development of teams able to effectively solve workplace problems - Managers able to make headway towards context-specific change - Some positive changes in health system performance (improved district HIS; improved support services from sub-districts to FMs and improved resourcing of PHC clinics)   Negative   - Resistance to change from other public sector managers/staff who had not gone through the programme - Mentorship relationships difficult to initiate or maintain | - Inability of public sector health/human resource departments to find a place for participants or graduates |
| Dovey 2002 | - Collaborative work-based projects - Coaching - 4 one week classroom lectures - 1 day Workshops - Reflection practices - District Health Management Certificate | Challenges   - Poor strategic organizational plans - Province underdeveloped and impoverished - Poor infrastructure within the province - Mismanagement, power struggles and corruption amongst senior management - organizational culture of entitlement within the Eastern Cape Department of Health   Opportunities   - Buy in across partners and the provincial government. All stakeholders were involved in designing, implementing and managing the intervention | General motivation in the workplace   - Perceive themselves powerless to impact their environment   Motivation directly linked to WPBL   - Establishment of core values/code of conduct critical factor to implementation of work-based projects - Achievement of goals garnered enthusiasm, commitment and confidence among DHMT members - Working collaboratively around shared mission and values, reduced mistrust and individual competiveness, thus becoming a source of motivation | Challenges   - Limited collaboration amongst public institutions due to history of apartheid - Poor preparation of leaders in public and private sector institutions | Positive   - Establishment of DHMT mission - Establishment of DHMT code of conduct - Setting realistic goals and action plans - Development of cohesive teams - Service delivery problems addressed through work-based projects e.g. supply of STI drugs to isolated clinics - Presentation of projects to senior managers | Positive   - DHMTs focused on achieving organizational goals - Development of procedural and strategic knowledge to solve complex workplace problems - Development of strategic thinking for goal setting - Development of social agency - Realization that workplace problems require shared positive collaboration, and sustained collaboration - Development of power management strategies - Built distributed leadership capacity in DHMTs   Negative   - Poor provincial leadership will put DHMTs capacity gains at risk - The focus of the programme was on the nature of learning gained (project report) and not whether the work-based projects succeeded or failed | - Work-based projects continued in successive cycles of strategic action as a new member of the DHMT enrolled in the programme. (Programme goal was to capacitate DHMT staff across the province each year.) |
| Edwards et al. 2015 | - Week long onsite mentoring every 4-8 weeks | Challenges   - Dearth health workforce - Poor district compliance with MOH accounting procedures (therefore delays in receiving funds) - High staff turnover - Poor resource allocation - Poor working conditions - Delays in and low pay - Staff away from work due to mandatory trainings and meetings - Lack of accountability - Frequent stock-outs of administrative forms - Limited or non-functioning transportation services - Poor documentation of status of district vehicles   Opportunities   - Willingness by provincial and district leaders to collaborate with partners | General motivation in the workplace   - Demotivated employees due to limited professional development | Challenges   - One of the poorest nations in the world - Poor road conditions   Opportunities   - Decentralized health system | Positive   - High-quality financial reports were prepared - Improvement in budget preparation to cover expenses like salaries - Payments were appropriately documented - Improvement in professional development for staff - Improvement in stock availability of administrative forms - Improved documentation of district vehicle usage - Improved scheduling of district vehicle maintenance | Positive   - Improved overall coordination and planning within the province - Greater transparency of accounting practices - Better district health financial resource allocation to meet community needs - Increase in the number of health workers - Improved forecasting of health system needs   Negative   - Increasing demand for health services adding an extra strain to ensure quality | - Management mentoring programme is within a vertically (HIV) financed intervention trying to achieve horizontal system-wide effect. - FGH gave sub-grants to each district to support programme - FGH directly hired healthcare workers for MOH. These workers were to be absorbed into MOH payroll in the subsequent fiscal year. Programme targeted accounting processes to ensure health workers were absorbed. - High human capital investment (mentors) needed which was time consuming and expensive. - Different planning and fiscal years between MOH and FGH (like staff away on mandatory national trainings and meetings thus not available during mentoring visits) - Constant changes to donor and MOH priorities. |
| Foster et al. 2018 | - Community health improvement project - Peer learning - Distance learning using a mobile application - In-person trainings - Support from supervisors who check-in monthly to facilitate discussions and review workbook exercises - Majority of the learning in the workplace engaging staff and community members with exercises integrated into service delivery activities - Certificate in Leadership and Management Practice | Challenges   - Under-staffed facilities - Heavy workloads - Limited access to technology - Nurses have no pre-service training in leadership and management - Difficulty assimilating and integrating CHAs into the community health team   Opportunities   - Key stakeholders engaged (UNZA, ZUNO, General Nursing Council and Health Professionals Council of Zambia.) | Motivation directly linked to WPBL   - Motivated to pursue training to earn CPD points for relicensure requirements - Facility heads gained the title of head nurse in charge on completion of training |  | Positive   - Facility heads strengthened their leadership and management skills - Improved relationships between facility heads and district managers - Nurses were able to build collaboration across different cadres like CHAs to address community needs - Improved communication between facility providers and the community - Participants gained experience and confidence in using technology like MS Office applications | Positive   - Proven progress in delivery, quality and accessibility of health services - Improved oversight and accountability of community health (due to joint participation of facility heads and district managers) - Built a network of support among nurse colleagues - Facility heads gained efficiency in delegation and task shifting to reduce heavy workloads - Health projects ensured responsiveness to community needs and requests - Programme built capacity across several members of the frontline PHC team (not just nurses) while implementing the health improvement projects - UNZA’s School of Nursing beginning to include elements of programme in the nursing pre-service training curriculum   Negative   - 2 FM’s did not complete the programme | - Approved as a national CPD programme and required for all nurses leading PHC rural facilities. - Country owned and community aligned (met priorities identified by the local health authority and ensured responsiveness to community needs.) - Programme complimented MOH guidelines and other MOH trainings - Made best use of resources (training design, delivery and tools) to ensure programme remained financial viable so that when external funding ended the programme would not end. - Programme development remained flexible to learn from previous cycles. |
| Gormley and McCaffery 2013 | - 3 one week workshops - Coaching - Action learning based on solving a specific problem at work - Systematic approach to programme planning (course structured around the six thematic action fields of the HRH Action Framework) - Facilitator actively involved in the learning process | Challenges   - High health worker absenteeism - Inadequate number and skills mix of health workforce   Opportunities   - Key stakeholders across districts and ministries involved in the design and delivery of the HRH development programme - HRH specialists attended programme as a team | Motivation directly linked to WPBL   - High commitment and buy-in to the programme |  | Positive   - Performance of staff on provisional status improved - Staff in acting positions promoted faster - HRH team able to identify root causes of high staff absenteeism - Teams able to collaborate together to solve HRH challenges | Positive   - Health workers responsive to community needs - Participants had positive attitudes towards the possibility of solving work challenges | - The resultant collaboration Ministry of Public Works and Health Services Commission ensured the taught management skills and practices would be maintained and sustained operationally; as well as guaranteeing that any organizational hindrances were resolved - Local country based facilitators were often those who had worked in district-level health facilities and therefore acquainted with district contexts and environments - LD intervention aligned with national and sub-national goals |
| Kwamie et al. 2014 | - Active role of facilitators (in workshops and coaching) - 2 day face-to-face workshops held in the capital city Accra 3 times bi-monthly - Monthly team coaching - Action projects around an identified MNH problem | Challenges   - Resources (human, diagnostic or financial) are constrained - District managers have time constraints due to managing both district and donor vertical programmes - Formalized management training is limited - District managers have more discretion in decision-making within donor vertical programmes | General motivation in the workplace   - Poor staff attitude   Motivation directly linked to WPBL   - Initially the novelty of the leadership development programme was received with positivity - Having regional health administration facilitate coaching enthused teams - Managers found the resource intensiveness of programme to be burdensome | Challenges   - GHS has a hierarchical structure where decision-making is highly centralized | Positive   - Managers better able to prioritize workload and concurrently manage vertical donor programmes - Managers able to support teamwork through inspiration and acknowledgment - Managers able on a small scale to build initiative towards solving problems   Negative   - Implementation of the programme exacerbated staff’s time and resource constraints | Positive   - Short-term service delivery outcomes included: Increased skilled birth delivery; reduced still births; and increased focused antenatal care   Negative   - The top-down manner in which the LD programme was introduced reinforced hierarchical, highly centralized, decision-making processes - No change in relationships between district and regional levels - No on-going coaching or mentorship post LD - Running external interventions that require planning, money and time (especially if not included in the planning cycles) exacerbated workloads and resource constraints | - Lack of LD programme institutionalisation due to routine district year end commitments and changes in regional and district leadership (dividing the district into two separate districts in October 2012 necessitated new administrative structures and a reorganization of relationships within the new districts.) - Managers never took ownership of the LD programme as they viewed it as a once off regional project (with no on-going follow through) versus a continuing district programme |
| Mansour et al.  2010 | - 4 one or two day workshops - service delivery improvement projects using MSH’s Challenge Model - Monthly support meetings led by MOH managers - Active role of facilitators (in workshops and meetings) - Some degree of structured planning to the programme (structured around MSH’s Leading and Managing Framework; led in Arabic by MOH facilitators; and programme exercises reviewed by the facilitators to ensure they were context-specific) |  | General motivation in the workplace   - Low morale among health workers and managers - Lack of commitment from front line health workers   Motivation directly linked to WPBL   - Participants enthusiastic of programme (enabled front line health workers to actively participate in design and implementation of their own improvement projects; and in the scale-up of the programme) - Programme’s hands-on approach promoted participants’ ownership of local health system. | Challenges   - Aswan Governorate mostly rural and improvised - Gaps in access to and quality of health services in lower resourced parts of the country | Positive   - Teams stopped complaining about problems; to identifying ones they could address - Teams able to analyse problems and use existing resources to address them - Teams learned to work together and mobilize other stakeholders to address public health problems | Positive   - All PHC facilities in Aswan have gone through the LD programme - 20 LD programme facilitators and participants have taken up senior health management positions in Aswan - Service providers have improved commitment and love for their jobs - Improved clients’ perceptions of health facilities and workers - Increased number of new family planning visits and use of contraceptives which reduced fertility rates - Increased prenatal care utilization - Reduced maternal mortality rate in Aswan 2 years after all PHC facilities had gone through the programme | - LD programme was taught in Arabic and led by MOH facilitators - Programme exercises reviewed by the MOH facilitators to ensure they were context-specific - Local managers taught the shorter workshop sessions as well as led district meetings between sessions to strengthen learning. - Due to sustainability and scale-up in Aswan, the LD programme has been implemented in other governorates in Egypt and in 35 countries in the global South |
| Martineau et al. 2018 | - 2 Workshops (1-2.5 days each) - Active role of facilitator throughout the MSI - Action learning sets - Reflection - Peer-learning through inter-district meetings - Systematic approach to programme planning (course structured around the action research cycle of: plan, act, observe and reflect) | Challenges   - Numerous changes in the leadership of one team in Tanzania led to a lack of continuity and undermined the development of management skills   Opportunities   - Workshops and inter-district meetings gave participants protected time to work through the action learning process - Willingness of DHMTs to participate in the MSI | Motivation directly linked to WPBL   - Fitted in with work schedule as workshops and meetings were short - Facilitation process encouraged and guided participants - Bringing districts together for workshops enabled peer-learning which participants appreciated | Opportunities   - Decentralized health system | Positive   - DHMTs able to design and implement integrated strategies to address district problems - DHMTS better able to allocate funds for district activities - Transformation in the way DHMTs functioned; such as ownership of problems; taking initiative; empowered to solve problems; and team work. - Improvement in management competencies of planning due to the deeper analysis of district problems and therefore better strategies to address them - Improved quality of supervision of front-line health workers | Positive   - Demonstrated improvements in service delivery (vaccine programme in Ghana) - Greater interaction between DHMTs and FMs (positive unintended consequence.)   Negative   - Unintended consequence of shortage of HIV drugs and equipment when demand for HIV services increased - Unintended consequence of neglect on service areas not targeted by the MSI | - Funds for implementation of work plans were deliberately not provided to instil an entrepreneurial approach to resource mobilization thus not jeopardize sustainability commonly seen once externally funded projects’ end. (This is a risk. Whether it actually works depends on each specific context.) Only successive MSI cycles can tell us if it works. |
| Mutale et al. 2017 | - Mentoring during and after training - 4 workshops (2-2.5 days each) - Action learning through working on solving problems in the workplace - Systematic approach to programme planning (logic model informed design of the ZMLA Programme) | Challenges   - Little time allocated for trainees to go through course work and participate in peer-learning - DHMTs run by recently qualified medical doctors with nominal leadership and management training | Motivation directly linked to WPBL   - Certified by NIPA - Training popular because it provided practical tools to address everyday health system challenges - Due to training trainees felt empowered and confident to be health system managers | Opportunities   - MOH developed a Governance and Management Capacity Building Strategic Plan (which led to adoption of in-service leadership and management training) | Positive   - Shared vision as teamwork and coordination improved - Improvements in attending meetings with a greater appreciation of their importance - Improved workplace climate particularly around human resource management - Trainees had greater role clarity and understanding of others roles’ and how this supported organizational goals - Trainees improved in financial literacy and thus improved in financial management | Positive   - Trainees remained in public health service - Trainees felt more prepared and motivated to lead and manage   Negative   - Many trainees did not implement the solutions generated to solve workplace problems as graduation did not depend on this | - After ZMLA received NIPA certification training became popular among busy health workers - Shortening programme length from 12 to 6 months (to reduce health service delivery disruptions); increased course workload but reduced attrition. - Questions of sustainability arose as the programme was a free donor funded course. (Offering it through a local institution could mitigate this issue.) |
| Nakanjako et al. 2015 | - 2 months didactic classroom modules - 9 months field attachment - Mentorship (during field attachment) - 4 online learning modules - Action projects | Challenges   - Many FMs do not have needed leadership or management training   Opportunities   - Institutions willing to allocate time for fellowship activities. (Action projects did not require funding but protected time to complete.) - Dedicated office space for fellows - Available projects for WPBL at attachment sites - Availability of organizational mentors | Motivation directly linked to WPBL   - Fellows empowered to assume more leadership responsibilities |  | Positive   - Improved competencies in leading and inspiring teams to generate results - Improved use of data to inform health service delivery | Positive   - All programme graduates stayed in health leadership positions within the country - New responsibilities given to fellows - Improved HIV/AIDS health service delivery - Overhaul of policy formation and review procedures; and health worker training in the national HIV/AIDS programme - Scale-up of some successfully implemented action projects - Joint structured mentorship (from Makerere and attachment sites) for a period of 10 months formed a foundation of trust and on-going support for development of action projects and leadership skills   Negative   - 1 fellow did not complete the programme | - Cost of programme was $40,000 to train 1 fellow each year (for fellow stipend, curriculum development, and travel to residential module trainings) |
| Nzinga et al. 2021 | - 5 day taught course on complex health systems - Course on emotional competence and communication skills - 3 day skill building course - Reflective practices - Systematic approach to leadership training (researchers and health system managers engagement cycles of planning, implementation and reflection of activities) - Action learning (addressing a health system problem using MSH’s Challenge Model) - Flipped classroom (using Kenyan case studies) - 2 and ½ day follow-up workshop | Challenges   - Deeply rooted cultures of not questioning authority (or their decisions on what needs to be given precedence in the health system) - Mistrust between meso-level managers and senior-level leaders - Lack of organizational practices to provide emotional support to employees   Opportunities   - Learning site (long-term partnership between health system managers and researchers to address heath system challenges) | General motivation in the workplace   - Frontline health workers apathetic to change   Motivation directly linked to WPBL   - Participants more confident at dealing with work problems and providing solutions - Managers found the LD programme valuable with learning useful to everyday work | Challenges   - Rapid devolution led to: unclear communication channels; reporting; and accountability - Political interference | Positive   - Better communication skills (brought about by learning to listen without interruption) - Managers able to engage and build their teams to collective address work problems - Managers began recognizing the importance of team members - Managers developed a growing ability to show respect and empathy and give constructive feedback | Positive   - Collaborative decision-making among teams - Better relationships with co-workers and subordinates - Resource mobilization during a nationwide nurses strike by engaging technical partners and senior leaders - Acquired new skills led to job satisfaction and increased work commitment   Negative   - Only 8 out of 30 participants went through the full training. - Providing feedback to superiors continued to be a problem as it was not appreciated - Lack of supportive networks prevented a number of managers from consistently attending the training | - The broader embedded nature of the RESYST LD team together with the participatory nature of co-developing the intervention with managers inspired learning, ownership of the LD intervention, as well as motivation to use skills learned in practice |
| Prashanth et al. 2014 (2) | - 2-5 contact classes/classroom teaching per month - Peer/group learning during classes - 5 on-site mentoring visits - Systematic approach to programme planning (course designed by a consortium of 5 NGOs with input from state government); and programme evaluated to assess whether learning outcomes were achieved | Challenges   - DHS or DHMTs concept not well defined/ established despite decentralization of health services being implemented for about a decade - High turnover of health managers - Under-staffed districts - Uncoordinated district health services with separate reporting lines to the state - Team power distance (dynamics) and prevailing socio-cultural values. (Doctors automatically viewed as team leaders despite the existence of non-medical management professionals/cadre.)   Opportunities   - District/State/Central levels’ openness to agree to change | General motivation in the workplace   - Frustration with decentralization planning as managers had no power to make changes - District health managers were apathetic and lacked desire to change things   Motivation directly linked to WPBL   - Some managers saw training as a way to achieve personal and organizational goals (seen by the differing outputs and outcomes of the LD programme within the same district) | Challenges   - Increasing political interference (district health services now accountable to PRI representatives) - Remote areas not a favoured posting preference by doctors   Opportunities   - Health system undergoing decentralization to district levels - NRHM implementation | Positive   - Marginal increase in financial resources being better utilized   Negative   - Poor teamwork | Positive   - Variable decreases in still birth rates across the district   Negative   - Managers were not able to always implement changes in the workplace often due to structural and resource constraints - Power distance between doctors and other team members remained - Health managers working in poorly resourced areas got frustrated by the lack of enabling action from higher levels of the health system | - Depended on whether workplace change suggestions were in line with central vision like NRHM implementation and local community needs. |
| Rowe et al. 2010 | - 3 classroom sessions (10 days each) - On-site mentoring - Active role of the facilitator (taught and mentored) - Action projects (around MCH, reproductive and adolescent health, controlling communicable disease, mental health, and emergency care) - Systematic approach to learning (programme structured around health systems management-problem solving, strategic thinking, HRH, and LD) | Challenges   - DHMTs had limited management training or experience.   Opportunities   - Started in 2007 on request from Mother Patern College |  | Challenges   - 14 years post-civil war   Opportunities   - Liberia transitioning from a time of needing relief and crisis management to one of growth and expansion - Decentralized health system - Endorsed by MOH; and as such MOH was a driving force in programme implementation. | Positive   - Improvement in management skills |  | - Over several cycles training responsibility was transferred to local facilitators while ensuring these facilitators/mentors were well trained. - Programme flexible to changes in course content and delivery methods while transferring to local facilitators ensuring programme ownership. - Use of a short course format to reduce work disruptions; focusing on a few vital skill areas with practical tools for application. - Limited turnover of Mother Patern College, Yale University and CHAI staff during transfer |
| Seims et al. 2012 | - Action learning (addressing a health system problem using MSH’s Challenge Model) - 4 LD workshops - Stakeholder alignment meetings with national and sub-national decision makers - Team coaching/ mentoring - Systematic approach to leadership training (training structured around MSH’s Leading and Managing Framework) | Challenges   - Staff shortages - Limited supply of medications and vaccines   Opportunities   - Willingness of DHMTs to participate in the LD intervention |  | Challenges   - Drought (some districts) - Insecurity (some districts) | Positive   - Improved work climate due to better staff training/ supervision; and repairing staff housing - Timely and accurate reporting of institutional service delivery data | Positive   - Significant increases in district health service coverage and number of facility client visits due to: - Increased community mobilization and health education - Increased outreach centres and increased service hours thus increasing access to health | - Positive health service delivery outcomes sustained for 6 months after the LD programme |
| Sherr et al. 2013 | - In-service training courses based on MOH curriculum on data-driven decision making - Quarterly post-training coaching from MOH supervisors - Mentorship from PHIT partnership teams embedded in the MOH at provincial level - Routine district meetings | Challenges   - Poor health service and quality - Underfunded district health systems - Extreme health worker shortage - Weak HIS - Heavy DHMTs workload - Chronic resource shortages   Opportunities   - PHIT institutions had more than 25 years collaborative experience in the country leading to a deep understanding of context |  | Challenges   - Sofala province poorest in the country - High disease burden   Opportunities   - High use of NHS - Health sector decentralization reforms ongoing | Positive   - Managers improved in collaborating with key stakeholders (PHIT partners) - Ownership of health system interventions (PHIT activities) by district and provincial leaders | Positive   - Better cooperation and engagement within the province to resolve health system challenges   Negative   - The extreme human resource constraint made it difficult to have personnel solely take on management positions - Difficult to define role and thus strengthen capacity of district managers due to slow and uneven decentralization process - Frequent turnover of district and provincial managers | - Shifts in national programmes and available funding required adaptations (flexibility) to the management and leadership training resulting in a focus on mentoring managers on formulating data driven decisions - Integrated into MOH provincial management structure to ensure sustainability and scalability. - Embedded nature of technical and financial assistance fostered ownership of management and leadership activities by health system leaders. - Focus on entire province to ensure sustainability of district capacity gains and what would be suitable for national scale-up. |
| Tetui et al. 2017 | - Systematic approach to management capacity building (structured around the PAR approach of problem identification, finding possible solutions, taking action and reflection) - Mentoring - Action project through MANIFEST - Quarterly review meetings for reflection and learning - Active role of facilitators in review meetings; and coordination and implementation of project | Challenges   - Staff shortages - Recurrent stock-out of medicines and supplies - Unsupportive work environment - FMs have heavy workloads - Laissez faire and working in isolation organizational culture - Excessive reliance on external organizations to carry out project implementation   Opportunities   - Willingness of district, sub-district and community level stakeholders to participate in the MANIFEST project activities | General motivation in the workplace   - Low health worker motivation - Low commitment to quality - Health workers rarely at work stations   Motivation directly linked to WPBL   - Involvement of different stakeholders led to motivation and a sense of ownership towards solving health system challenges | Challenges   - Poorly resourced local governments - Poor health outcomes in rural areas - Poor cooperation among government agencies and different administrative levels     Opportunities   - Decentralized health system | Positive   - Improved teamwork and collaboration with stakeholders - Empowered and actively engaged community stakeholders - Free and open dialogue which led to trust amongst district teams and stakeholders - Better utilization of existing resources - Improved allocation of financial resources - Managers increased their confidence in decision-making; planning and coordinating district activities; and reporting and utilizing health data | Positive   - Free and open dialogue (reducing power dynamics) led to trust amongst district teams and stakeholders; leading to commitment towards project goals to improve maternal and neonatal health outcomes - Stakeholder collaborations increased managers’ awareness of and adaptability to community needs despite the desire to maintain the status quo - Construction of health facility infrastructure projects - Ambulances bought and/or donated - Improved health workers attitude - Reduced health workers absenteeism   Negative   - Excessive reliance on external organizations (local partner) to carry out project implementation | - Intervention implemented using existing district structure and resources to increase chances of institutionalization and sustainability - There was district level commitment that enabled and undergirded cooperation among stakeholders with differing power. - Flexibility and wide stakeholder engagement enhanced local creativity and innovation to meet community needs. |
| Tomblin Murphy et al. 2022 | - On-site mentoring and coaching between workshops - 2 face-to-face 5-day capacity building workshops in 2018 and 2020 at the Tanzania Training   Centre for International Health in Ifakara   - Systematic approach to the leadership and management capacity building initiative - 4 eLearning modules - Action learning [addressing major leadership and management gaps that affected productivity (provision of care and outcomes)] | Challenges   - Gap in leadership and   managerial capacity at PHC facilities and among CHMTs - lack of prior leadership training before attaining leadership position   - Lack of financial resources - Limited facility staffing - Insufficient implementation   of maternal health interventions | Motivation directly linked to WPBL  Improved morale among health care providers and CHMT members. | Challenges   - Ineffective MNH leadership and governance | Positive   - Improved health facility management, use of data for planning, staff performance assessment, organization   of services, handling of emergencies and referrals, and  infection prevention and control.   - Improved team climate - Enhanced teamwork among health care providers and CHMT members - Improved accountability to the community - Role clarity increased | Positive   - Improved patient outcomes - New leadership roles attained - Teams highly committed to work collaboratively - Overall job satisfaction increased - Improved quality of MNH services | - Strengthening leadership and management skills at various levels of the health system (RHMT, CHMT, and FMs) fostered an environment that supported shared decision-making, accountability, and enhanced performance within PHC facilities. - The participation of district quality improvement coordinators in the national star-rating program was vital for ensuring the initiative's sustainability at the district level and possible national scalability. |
| **Key:** CHAs – Community Health Assistants; CHMTs – Council Health Management Teams; CPD – Continuous Professional Development; DHMTs – District Health Management Teams; DHS – District Health System; FGH - Friends in Global Health; FMs – facility managers; GHS – Ghana Health Service; HIS – health information systems; HIV/AIDS – Human Immunodeficiency Virus/Acquired Immunodeficiency Syndrome; HMIS – Health Management Information System; HRH – Human Resources for Health; LD – leadership development; MANIFEST – Maternal and Neonatal Implementation for Equitable Systems; MCH – Maternal and Child Health; MNH – Maternal and New-born Health; MOH – Ministry of Health; MSH – Management Sciences for Health; MSI – Management Strengthening Intervention; NGOs – Non-governmental organisations; NHS – National Health Service; NIPA – National Institute for Public Administration; NRHM – National Rural Health Mission (healthcare reforms to improve financing, planning and supervision of health); PAR – Participatory Action Research; PHC – primary healthcare; PHIT – Population Health Implementation and Training; PRI – Panchayati Raj Institution (elected members of local government); RESYST – Resilient and Responsive health systems; RHMT – Regional Health Management Team; SDMT – Sub-District Management Team; STI – Sexual Transmitted Infections; UNZA – University of Zambia; USAID – United States Agency for International Development; WPBL – Workplace based learning; ZMLA – Zambia Management and Leadership Academy; ZUNO – Zambian Union of Nurses Organization | | | | | | | |

**Appendix 4.** Adapted workplace based learning conceptual framework (Matthews, 1999; Day, 2001; Cunningham et al., 2004; WHO, 2007; Raelin, 2008; Jacobs and Park, 2009; Manley et al., 2009; Vriesendorp et al., 2010; Doherty and Gilson, 2015)


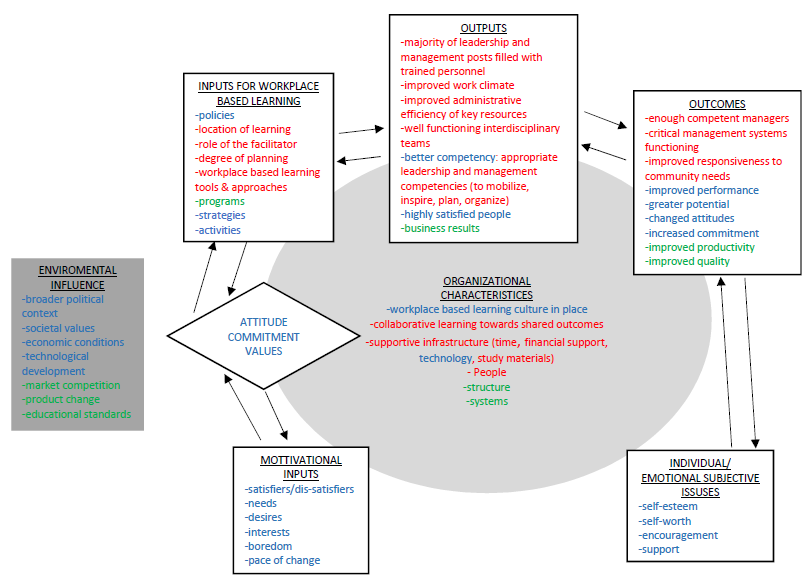


**Key:**

1. Green highlighted words show concepts from the original Matthews (1999) framework that were removed for this review.
2. Blue highlighted words show concepts from the original Matthews (1999) framework that remained and were applicable for this review.
3. Red highlighted words show concepts that were added. The framework, peer-reviewed literature or book from which these concepts were derived from is shown in the table below.

| Framework/Peer-reviewed literature/ book | Inputs for WPBL | Organizational  Characteristics | Outputs | Outcomes |
| --- | --- | --- | --- | --- |
| Cunningham et al. 2004 | Workplace based learning tools & approaches (Action Projects, Acton Learning, Mentoring, Coaching & Reflection) |  |  |  |
| Day 2001 | Workplace based learning tools & approaches (Acton Learning, Mentoring, Coaching & Peer-learning) |  |  |  |
| Doherty and Gilson 2015 | Workplace based learning tools & approaches (Facilitators, Action Projects, Acton Learning, Mentoring, Coaching, Peer-learning, Reflection & Case Studies) |  |  |  |
| Jacobs and Park 2009 | - Location of learning - Role of the facilitator - Degree of planning |  |  |  |
| Manley et al. 2009 |  | - Collaborative learning towards shared outcomes - Supportive infrastructure (time, financial support, study materials) | Well-functioning interdisciplinary teams |  |
| Raelin 2008 | Workplace based learning tools & approaches (Acton Learning, Peer-learning & Reflection) |  |  |  |
| Vriesendorp et al. 2010 |  |  | Appropriate leadership and management competencies (to mobilize, inspire, plan, organize) |  |
| WHO 2007 |  |  | - Improved administrative efficiency of key resources - Majority of leadership and management posts filled with trained personnel - Improved work climate | - Enough competent managers - Critical management systems functioning - Improved responsiveness to community needs |
