# Supplementary material for: Workplace-based learning in district health leadership and management strengthening: a framework synthesis
Source: Health Policy Plan. 2024 Oct 9;40(1):105–19. doi: 10.1093/heapol/czae095 (PMC11724643; doi:10.1093/heapol/czae095)
Supplement: czae095_Supp [file czae095_supp.zip › czae095_Supp/Table1.docx]

**Table 1.** Inclusion and exclusion criteria

| Inclusion Criteria | Exclusion Criteria |
| --- | --- |
| - Studies relevant to the research question - Empirical studies on WPBL linked to leadership and/or management development - Studies that include qualitative, mixed-methods and quantitative methodology - Studies published in English - Grey literature in English from credible sources like WHO or AHPSR - Studies carried out in LMICs - Studies carried out in PHC facilities as well as at the district level - Original or review articles whose titles and abstracts include one or more of the key search terms | - Studies not linked to WPBL or applicable to the research question |
|  | - Studies which are not based on empirical research |
|  | - Studies not published in English |
|  | - Studies not carried out in LMICs |
|  | - Studies published before the year 1990 |
|  |  |
|  |  |
|  |  |
