# Supplementary material for: Workplace-based learning in district health leadership and management strengthening: a framework synthesis
Source: Health Policy Plan. 2024 Oct 9;40(1):105–19. doi: 10.1093/heapol/czae095 (PMC11724643; doi:10.1093/heapol/czae095)
Supplement: czae095_Supp [file czae095_supp.zip › czae095_Supp/Table2.docx]

**Table 2.** Number of articles from each country

| Country | Number of articles |
| --- | --- |
| Kenya | 6 |
| South Africa | 4 |
| Uganda | 4 |
| Mozambique | 2 |
| Zambia | 2 |
| India | 2 |
| Ghana | 2 |
| Liberia | 1 |
| Tanzania | 2 |
| Egypt | 1 |
| Ethiopia | 1 |
