# Supplementary material for: Workplace-based learning in district health leadership and management strengthening: a framework synthesis
Source: Health Policy Plan. 2024 Oct 9;40(1):105–19. doi: 10.1093/heapol/czae095 (PMC11724643; doi:10.1093/heapol/czae095)
Supplement: czae095_Supp [file czae095_supp.zip › czae095_Supp/Table3.docx]

**Table 3.** Types of methodologies utilized in the included studies

| Study Methodology | Number of Studies |
| --- | --- |
| Qualitative study^^[[1]](#footnote-1)^^ | 13 |
| Mixed-methods study | 4 |
| Quasi-experimental study | 4 |
| Quantitative study | 4 |

1. In this review a qualitative study was a study that only employed qualitative methods and data was collected through interviews, participant and non-participant observations, focus groups and document reviews (Tolley et al., 2004). [↑](#footnote-ref-1)
