# Supplementary material for: Workplace-based learning in district health leadership and management strengthening: a framework synthesis
Source: Health Policy Plan. 2024 Oct 9;40(1):105–19. doi: 10.1093/heapol/czae095 (PMC11724643; doi:10.1093/heapol/czae095)
Supplement: czae095_Supp [file czae095_supp.zip › czae095_Supp/Table4.docx]

**Table 4.** Stakeholders driving WPBL interventions

| Stakeholders driving WPBL | Number of interventions |
| --- | --- |
| Country level higher education institutions | 10 |
| Country level research institutions | 2 |
| Other country level actors | 5 |
| External actors and/donors | 3 |
| Collaborations between country level actors and external actors/donors | 7 |
