# Supplementary material for: Workplace-based learning in district health leadership and management strengthening: a framework synthesis
Source: Health Policy Plan. 2024 Oct 9;40(1):105–19. doi: 10.1093/heapol/czae095 (PMC11724643; doi:10.1093/heapol/czae095)
Supplement: czae095_Supp [file czae095_supp.zip › czae095_Supp/Table5.docx]

**Table 5.** Participants involved in WPBL interventions

| WPBL Participants | Number of interventions |
| --- | --- |
| District Managers or DHMT(s) | 14 |
| Facility Managers | 13 |
| Sub-District Management Team(s) | 4 |
| Health Workers | 5 |
| Elected members of local government | 1 |
| Interdisciplinary participants involved in district financial management | 1 |
| District Human Resources for Health teams | 1 |
| District Hospital Management Team | 1 |
| Other Staff in PHC facilities | 1 |
| Provincial (Governorate/Regional) Managers | 2 |
