# Supplementary material for: Workplace-based learning in district health leadership and management strengthening: a framework synthesis
Source: Health Policy Plan. 2024 Oct 9;40(1):105–19. doi: 10.1093/heapol/czae095 (PMC11724643; doi:10.1093/heapol/czae095)
Supplement: czae095_Supp [file czae095_supp.zip › czae095_Supp/Table6.docx]

**Table 6.** Inputs of WPBL interventions utilized for leadership and management development

| WPBL Inputs | Number of Interventions |
| --- | --- |
| Facilitators | 19 |
| Action Projects | 10 |
| Mentoring | 10 |
| Coaching | 9 |
| Peer-learning | 7 |
| Acton Learning | 7 |
| Reflection | 6 |
| Case studies | 3 |
